# Synthesis, oligonucleotide incorporation and fluorescence properties in DNA of a bicyclic thymine analogue

**DOI:** 10.1038/s41598-018-31897-2

**Published:** 2018-09-18

**Authors:** Christopher P. Lawson, Anders F. Füchtbauer, Moa S. Wranne, Tristan Giraud, Thomas Floyd, Blaise Dumat, Nicolai K. Andersen, Afaf H. El-Sagheer, Tom Brown, Henrik Gradén, L. Marcus Wilhelmsson, Morten Grøtli

**Affiliations:** 1 Department of Chemistry and Molecular Biology, University of Gothenburg, S-41296 Gothenburg, Sweden; 2 Department of Chemistry and Chemical Engineering, Chemistry and Biochemistry, Chalmers University of Technology, Gothenburg, SE-412 96 Sweden; 3 Department of Chemistry, University of Oxford, Chemistry Research Laboratory, 12 Mansfield Road, Oxford, OX1 3TA UK; 4 Chemistry Branch, Department of Science and Mathematics, Faculty of Petroleum and Mining Engineering, Suez University, Suez, 43721 Egypt; 5 Cardiovascular, Renal and Metabolic Diseases IMED Biotech Unit, AstraZeneca Gothenburg, Pepparedsleden 1, Molndal, SE-431 83 Sweden

## Abstract

Fluorescent base analogues (FBAs) have emerged as a powerful class of molecular reporters of location and environment for nucleic acids. In our overall mission to develop bright and useful FBAs for all natural nucleobases, herein we describe the synthesis and thorough characterization of bicyclic thymidine (bT), both as a monomer and when incorporated into DNA. We have developed a robust synthetic route for the preparation of the bT DNA monomer and the corresponding protected phosphoramidite for solid-phase DNA synthesis. The bT deoxyribonucleoside has a brightness value of 790 M^−1^cm^−1^ in water, which is comparable or higher than most fluorescent thymine analogues reported. When incorporated into DNA, bT pairs selectively with adenine without perturbing the B-form structure, keeping the melting thermodynamics of the B-form duplex DNA virtually unchanged. As for most fluorescent base analogues, the emission of bT is reduced inside DNA (4.5- and 13-fold in single- and double-stranded DNA, respectively). Overall, these properties make bT an interesting thymine analogue for studying DNA and an excellent starting point for the development of brighter bT derivatives.

## Introduction

Intrinsic and extrinsic fluorophores including fluorescent base analogues (FBAs) are proven to be vital tools with wide-ranging applications in biology and biotechnology as molecular probes, reporters and labels for nucleic acids^1–4^. For example, they facilitate precise, real-time tracking of labelled components in a living system^5^. Over the last two decades, a multitude of fluorescent nucleobase moieties have been synthesized^6–8^. Initially, this involved the attachment of fluorescent labels to native nucleosides *via* a non-emissive linker, which allowed them to report on *e*.*g*. changes in the microenvironment around nucleic acids^9^. However, since these fluorophores generally protrude significantly from the nucleic acid structure, they may interfere with the mobility and geometry of their hosts, perturbing the delicate biochemical balance critical for optimal biological function. An alternative approach involves the design and synthesis of modified nucleosides with intrinsic fluorescence. These FBAs can mimic the shape and hydrogen-bonding ability of the natural nucleobases and can be incorporated directly into DNA- or RNA-strands, chemically or enzymatically, often causing minimal perturbation of the nucleic acid structure^6–8^. Their location within the nucleic acid structure ensures that they report on the properties of the nucleic acid architecture under investigation rather than on their own intrinsic dynamics (with a few exceptions such as 2-AP)^10^. However, the size, shape and base-pairing restrictions imposed on them as analogues of the natural nucleobases make it difficult to improve the brightness and significantly modulate their excitation and emission to longer wavelengths.

**Figure 1:**
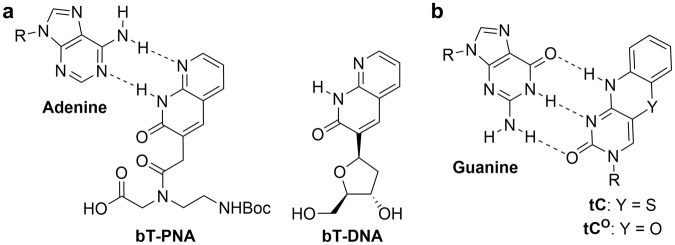
(**a**) The structure of the PNA- and DNA-derivative of bicyclic thymine (bT), base-paired with adenine. (**b**) The structure of tC/tC°, base-paired with guanine. R denotes the sugar-phosphate backbone.

While there are now several examples of adenine and cytosine analogues that show high brightness within nucleic acids, bright examples of thymine FBAs are less common. In reports by Eldrup *et al*., a series of 1,8-naphthyridin-2(1 *H*)-ones were presented as novel bicyclic (bT, Fig. 1a, left) and tricyclic (tT) analogues of thymine, some of which were found to be more efficient than thymine in the recognition of adenine in peptide nucleic acids (PNAs) duplex and triplex structures^11,12^. A preliminary photophysical analysis of these thymine PNA monomers revealed similarities (data not shown) to the bright and stable donors tC and tC°, which we previously reported, for instance, as part of the first FBA FRET-pair (Fig. 1b)^13–15^.

Pursuant to our interest in the development and characterization of FBAs for each natural nucleobase, we here report the synthesis and incorporation into DNA of the deoxyribose-derivative of bT (Fig. 1a, right), and characterize its base-mimicking and fluorescence properties inside DNA. We have recently shown that the photophysical properties of the excellent, but not so bright, adenine analogue, quadracyclic adenine (qA)^16^, was significantly improved by minor alterations to the scaffold, producing the bright and useful analogues qAN1 and pA^17–19^. We therefore envision that the bT scaffold will serve as an excellent starting point for the development of similarly bright thymine analogues with interesting photophysical properties.

## Results and Discussion

### Synthesis of the bT deoxyribonucleoside

**Figure 2:**
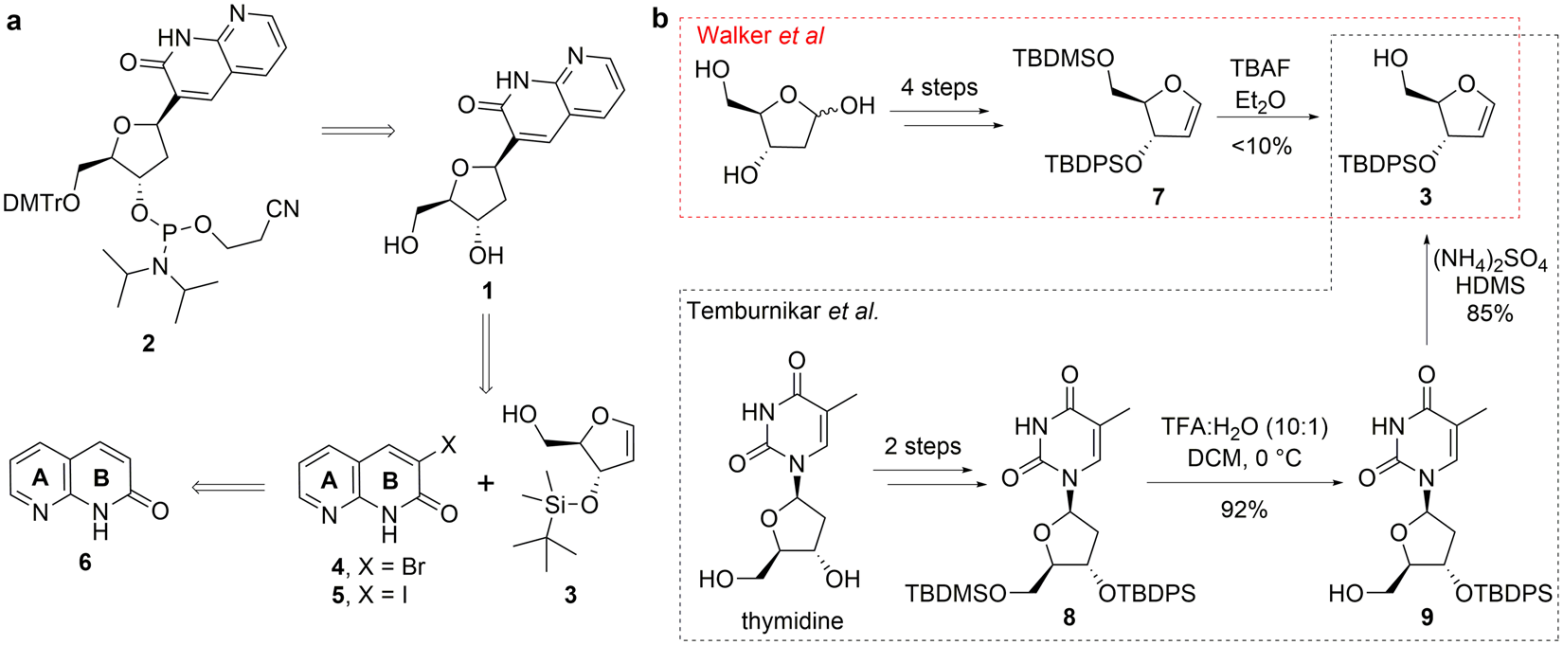
(**a**) Retrosynthetic analysis. (**b**) Synthetic routes to the glycal **3**^20,21^.

Unlike the PNA monomer of bT reported by Eldrup *et al*.^11^, a deoxyribose bT analogue (**1**, Fig. 2a) has no linker to the naphthyridinone core. We therefore envisioned that the target molecule (**2**) required for oligonucleotide synthesis could be obtained from a Heck coupling reaction between the protected glycal (**3**) and a suitably activated halo-naphthyridinone, such as **4** or **5** (Fig. 2). The halo-naphthyridinone could be prepared from commercially available 1,8-naphthyridin-2(1 *H*)-one (**6**).

Several reports for the synthesis of glycals exist in the literature (Fig. 2b)^20–22^. An initial attempt was made to synthesise **3**, utilising the protocol reported by Walker *et al*^22^. However, the di-protected glycal **7** was only obtained in moderate yield. Low yields had been reported for small-scale attempts on the subsequent selective deprotection of the 1° alcohol^22^, but in our experiments, the product was only isolated in very poor yields (<10%), even upon scale-up of the reaction, with the fully deprotected glycal being the major component of the reaction mixture. The glycal **3** was eventually synthesised utilising a modified version of the protocol reported by Temburnikar *et al*. (Fig. 2b) starting from thymidine^21^. In our experiments, the selective deprotection of the 1° alcohol **8** utilising BF_3_ etherate:TBAF (1:1) described in the original report gave low yields with high variability. However, running the reaction at 0 °C in TFA:water (10:1) for 4 hours was reliable and reproducible, yielding the desired compound **9** in excellent yield (92%). Treatment of **9** with ammonium sulphate in HDMS generated glycal **3** in a very good yield (85%).

Initial attempts to synthesise the 3-halo-1,8-naphtyridin-2(1 *H*)-one required as the coupling partner for the proposed Heck reaction by direct iodination of 1,8-naphthyridin-2(1 *H*)-one (**6**, Fig. 2) to afford 3-iodo-1,8-naphthyridin-2(1 *H*)-one (**5**) were unsuccessful. This was also the case for attempts to construct the B ring with the halogen in place *via* condensation reactions or by ring-closing metathesis protocols. 3-Bromo-1,8-naphthyridin-2(1 *H*)-one (**4**) was eventually obtained from a modification of a previously reported method used in the direct conversion of the commercially available 3-carboxylic acid derivative (**10**) to the corresponding bromide (Table 1)^23^. In our hands, the reported conditions resulted in a complex mixture containing approximately 20% of **4**, as indicated by UPLC-MS/UV analysis, along with a similar amount of di-brominated product (**4a**). Interestingly, changing the solvent to THF resulted in a very clean reaction to **4a**, thus providing a useful entry into 6-substituted bT-derivatives (Table 1). Following a process of enhancements to improve the isolated yields of the desired product (Table 1), **4** was efficiently and reproducibly isolated in quantitative yield and excellent purity following precipitation from the reaction mixture.

**Table 1:** Summary of the enhancement process.


Conditions	T (°C)	Conversion to 4 (UPLC-MS/UV)	Comments
Py/DMF/Br_2_ (10 equiv.)	105	~20%	Complex mixture including **4** and **4a**
Py/THF/Br_2_ (10 equiv.)	100	0%	Exothermic; **4a** only
Py/THF/Br_2_ (2 equiv.)	100	~30%	**4** and **10** only
DABCO/THF/Br_2_ (2 equiv.)	100	0%	
DMAP/H_2_O/K_2_CO_3_/Br_2_ (2 equiv.)	100	0%	Decarboxylation
DMAP/THF/DABCO/Br_2_ (2 equiv.)	100	—	Complex mixture of products
DMAP/THF/Br_2_ (2 equiv.)	100	—	Complex mixture of products
Py/THF/DMAP (20 mol%)/BBr_3_ (20 mol%)/Br_2_ (2 equiv.)	80	97%	Clean conversion to **4**

**Figure 3:**
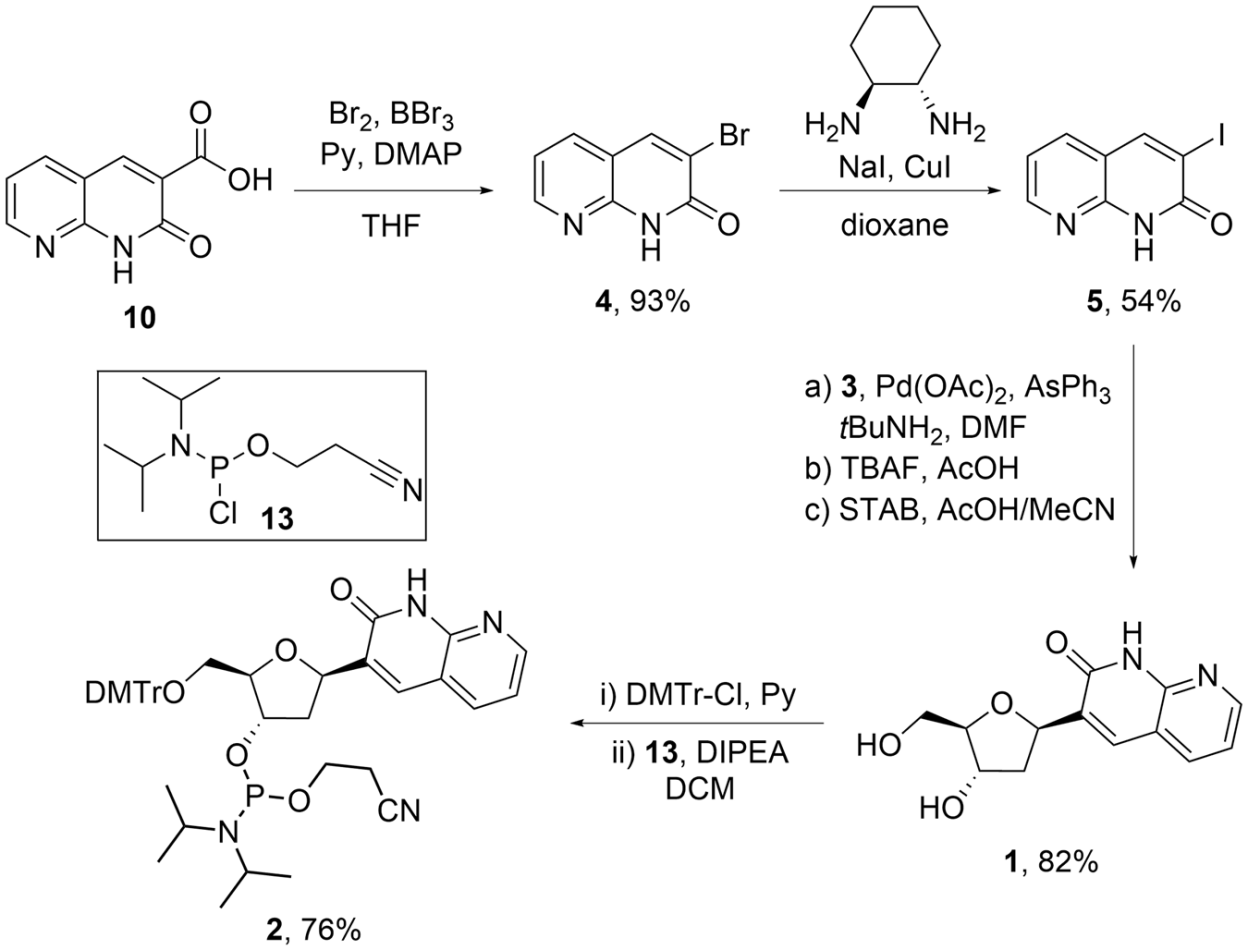
Synthesis of the phosphoramidite nucleoside of bT (**2**).

Employing **4** directly in the Heck reaction afforded **1** in a low yield (30%), after a challenging and laborious purification. Trans-halogenation by the protocol reported by Klapars *et al*., however, afforded **5** in a moderate yield (54%)^24^. Utilising **5**, **1** was obtained in good yield (82% following purification by HPLC) *via* a two-step process involving a Heck reaction followed by reduction (Fig. 3). Compound **1** was DMTr-protected and converted to the phosphoramidite monomer of bT (**2**) using standard procedures. We envision that this synthetic route should be capable of accommodating simple variations in the substitution pattern of bT, thus allowing the rapid generation of bT-derivatives from **10** or derivatives thereof.

### Photophysical properties of the bT deoxyribonucleoside

**Figure 4:**
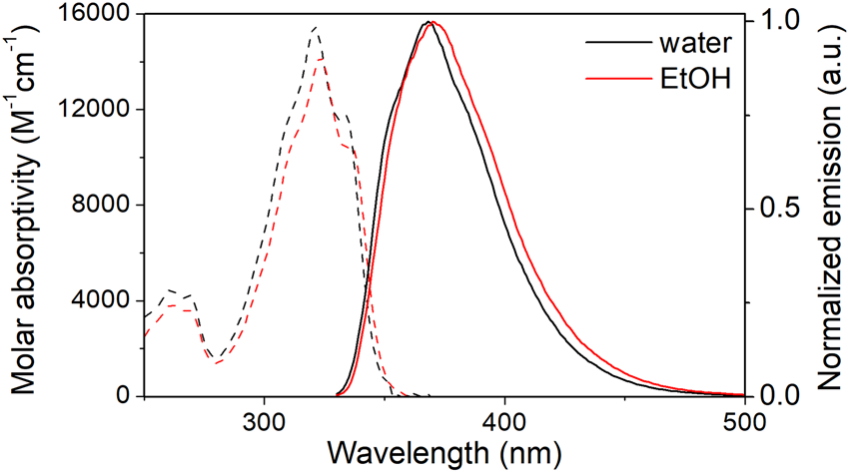
Molar absorptivity (dashed) and normalized fluorescence (solid) spectra of the bT deoxyribonucleoside (**1**) in water and ethanol.

The photophysical properties of the bT deoxyribonucleoside (**1**) were examined prior to incorporation into oligonucleotides. The absorption spectra of bT in water and ethanol are characterized by a structured long-wavelength peak at 321 and 323 nm respectively, with a molar absorptivity of around 15000 M^−1^ cm^−1^ in both cases (Fig. 4). The emission spectra of the bT nucleoside show a less structured single emission peak with a maximum at 368 nm in water and 370 nm in ethanol, respectively. The quantum yield of bT is 5.1% in water and 5.9% in ethanol, resulting in brightness (ε·Φ_F_) values of 790 and 840 M^−1^ cm^−1^, respectively. This is comparable to or higher than the values reported for most thymidine/uridine analogues in water, such as 5-(furan-2-yl)-2′-deoxyuridine (ε·Φ_F_ = 330)^25^, ^DMA^T (ε·Φ_F_ = 87)^26^, xT (ε·Φ_F_ = 1020 in MeOH)^27^ or the pyrenyl-deoxyuridines (1PydU and 2PydU; ε·Φ_F_ ≈ 500 in MeOH)^28^, but lower than a few bright thymine analogues, such as, ^th^U (ε·Φ_F_ = 1300)^29^, FCU (ε·Φ_F_ = 4800)^30^ and BgQ (ε·Φ_F_ = 12300)^31^.

### Incorporation of bT into DNA oligonucleotides

To study the effect on DNA structure and stability when thymine is replaced by bT, 16 bT-modified sequences were synthesized, wherein bT was flanked by all combinations of neighbouring bases (Table 2). The corresponding unmodified and complementary sequences were also synthesized, as well as sequences for a mismatch study. For details of the solid-phase oligonucleotide synthesis, see the Supporting Information.

**Table 2:** Melting temperatures of bT-modified duplexes (*T*_m_^bT^), unmodified duplexes (*T*_m_^T^), and the difference (Δ*T*_m_) between them.

Sequence name^a^	DNA sequence^b^	*T*_m_^bT^ (°C)	*T*_m_^T^ (°C)	Δ*T*_m_ (°C)
AA	5′-d(CGCA**A**(bT)**A**TCG)-3′	41.5	41.8	−0.3
AC	5′-d(CGCA**A**(bT)**C**TCG)-3′	46.2	47.0	−0.8
AG	5′-d(CGCA**A**(bT)**G**TCG)-3′	47.7	48.8	−1.1
AT	5′-d(CGCA**A**(bT)**T**TCG)-3′	42.9	42.4	0.5
CA	5′-d(CGCA**C**(bT)**A**TCG)-3′	45.8	46.4	−0.6
CC	5′-d(CGCA**C**(bT)**C**TCG)-3′	49.5	50.7	−1.2
CG	5′-d(CGCA**C**(bT)**G**TCG)-3′	51.0	52.5	−1.5
CT	5′-d(CGCA**C**(bT)**T**TCG)-3′	47.3	47.9	−0.6
GA	5′-d(CGCA**G**(bT)**A**TCG)-3′	44.3	44.8	−0.5
GC	5′-d(CGCA**G**(bT)**C**TCG)-3′	50.9	51.4	−0.5
GG	5′-d(CGCA**G**(bT)**G**TCG)-3′	51.0	51.0	0.0
GT	5′-d(CGCA**G**(bT)**T**TCG)-3′	47.8	48.0	−0.2
TA	5′-d(CGCA**T**(bT)**A**TCG)-3′	42.8	42.7	0.1
TC	5′-d(CGCA**T**(bT)**C**TCG)-3′	46.6	46.4	0.2
TG	5′-d(CGCA**T**(bT)**G**TCG)-3′	48.1	48.2	−0.1
TT	5′-d(CGCA**T**(bT)**T**TCG)-3′	45.4	45.5	−0.1

^a^Sequences are named by the bases neighbouring bT on the 5′- and 3′-sides, respectively. ^b^Unmodified samples contain a thymine instead of bT. Duplexes were formed by hybridization with the complementary strand as described in the experimental section. The melting temperatures were calculated as the maximum of the first derivative of the UV-melting curves, with a standard error of ≤0.6 °C. For individual error values, see Table S1.

### Conformation and stability of bT-modified duplexes

Circular dichroism (CD) analysis of the 16 modified and unmodified strands annealed with their complementary sequences shows that all bT-modified duplexes exhibit the archetypal characteristics of B-form DNA, *i*.*e*. positive bands at 260 and 280 nm and a negative band around 245 nm (Figures S1 and S2), suggesting that bT-modified duplexes adopt a normal B-form geometry^32^. There are minor differences between CD-spectra of modified and unmodified duplexes, but these most likely originate from differences in the absorption spectra between bT and thymine. Interestingly, the long-wavelength absorption band of bT was only observed in the CD spectra of duplexes where cytosine flanks bT on the 5′-side (Figure S3). For some base analogues, such as tC and 2-AP, the long-wavelength absorption band is observed in CD^33,34^, whereas for others, such as qA or tC°, no such band is observed^16,35^. Base analogues where the appearance of the long-wavelength absorption band is dependent on the surrounding bases are rare. Further studies of bT may therefore help shed light on the molecular basis of this induced CD in nucleobase stacks – a phenomenon that is still not fully understood.

The thermal stabilities of the bT-modified and the corresponding unmodified DNA duplexes are summarized in Table 2. Overall, incorporation of bT has a negligible effect on the stability of the duplex, and on average decreases the stability by only 0.4 °C, a desirable feature that is relatively uncommon among FBAs, although a few thymine analogues have been shown to leave the melting temperature essentially unchanged when incorporated into a duplex sequence^25,36^.

**Figure 5:**
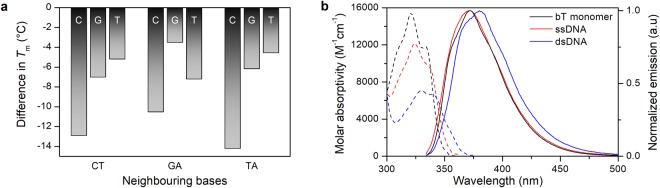
(**a**) Decrease in melting temperature upon base pairing bT with cytosine, guanine or thymine instead of adenine for three different sets of nearest bT neighbours (CT, GA and TA). The melting temperatures were calculated as the maximum of the first derivative of the UV-melting curves with a standard error of ≤0.6 °C. (**b**) Molar absorptivity (dashed) and normalized fluorescence (solid) spectra of the bT monomer (**1**) in water (black), bT-containing ssDNA (TT, red) and bT-containing dsDNA (TT, blue). Measurements were performed in phosphate buffer, pH 7.4, 150 mM Na^+^.

Thermal stability was also measured for mismatched sequences where bT is positioned opposite either cytosine, guanine or thymine instead of the matching adenine. This was done for three different nearest bT neighbours, GA (only purines), CT (only pyrimidines) and TA (mix) and the results are shown in Fig. 5a. All mismatches lower the melting temperature considerably, indicating that bT is selective towards adenine. A mismatch with cytosine lowers the melting temperature by 12.5 °C on average, while guanine and thymine mismatches both give an average decrease of 5.6 °C. This is consistent with previous observations that the dual pyrimidine mismatch (C-T) is particularly unfavourable^37–39^.

### Photophysical properties of bT inside DNA

Representative absorption and emission spectra of bT in single- and double-stranded DNA (ssDNA and dsDNA, respectively) are shown in Fig. 5b, with the corresponding spectra measured for the bT deoxyribonucleoside in water. The quantum yields of bT inside DNA are reported in Table 3, together with the wavelength of the lowest energy absorption and emission maximum. In DNA, the absorption maximum of bT is slightly red-shifted compared to the monomer (on average 325 nm and 329 nm for ssDNA and dsDNA respectively, compared to 321 nm for the deoxyribonucleoside). The wavelength of the lowest energy emission maximum is mildly sensitive to neighbouring bases, with average values of 369 and 375 nm for ssDNA and dsDNA, respectively. The molar absorptivity of the lowest energy transition varies slightly with neighbouring bases with average values of 11500 and 6200 M^−1^ cm^−1^ in ssDNA and dsDNA, respectively. Hypochromicity is seldom reported for fluorescent base analogues. However, when comparing a monomeric chromophore to the same chromophore inside duplex DNA, hypochromicities of 10–50% are frequently observed, and, consequently, the 58% decrease in molar absorptivity for bT is high, but not remarkable^40^. The hypochromicity can be attributed to strong stacking interaction effects between bT and the surrounding nucleobases when forming single- and double-stranded DNA.

**Table 3:** Photophysical properties of bT in the 16 modified oligonucleotides in single- (ssDNA) and double-stranded (dsDNA) environment.

Sample^a^	ssDNA			dsDNA		
	λ_Abs_ (nm)	λ_Em_ (nm)	Φ_F_^b^ (%)	λ_Abs_ (nm)	λ_Em_ (nm)	Φ_F_^b^ (%)
AA	326	369	0.4	328	377	0.4
AC	325	369	0.9	329	376	0.3
AG	326	367	0.2	329	379	0.1
AT	326	369	0.7	328	380	0.6
CA	325	369	1.0	328	372	0.3
CC	324	370	2.2	330	374	0.3
CG	326	368	0.3	330	378	0.1
CT	324	371	2.2	329	374	0.3
GA	327	368	0.2	327	372	0.1
GC	323	369	2.4	330	372	0.1
GG	326	368	0.2	326	372	0.1
GT	325	369	0.5	330	372	0.2
TA	325	369	1.0	329	377	0.9
TC	325	370	1.9	329	377	0.5
TG	324	368	0.3	328	373	0.1
TT	324	370	2.6	330	380	1.5

^a^For sequences, see Table 2. Measurements were performed at room temperature in phosphate buffer, pH 7.4, 150 mM Na^+^, 12.5 mM phosphate. ^b^Quantum yields were measured with quinine sulphate as reference (Φ_F_ = 54.6% in 0.5 M H_2_SO_4_). The reported values have a standard error ≤ 0.1% and are the averages of two or more measurements.

The quantum yield of bT varies substantially depending on the neighbouring bases. Overall, bT exhibits an average quantum yield of 1.1% in ssDNA and 0.4% in dsDNA, corresponding to 4.5-fold and 13-fold quenching, respectively. The highest quantum yield in dsDNA (1.5%) is obtained for TT, with thymines as 3′- and 5′-neighbours. From Table 3 it can be concluded that thymine, in general, is the least quenching neighbour while guanine is the most quenching one. Quenching by guanine has been observed for many other FBAs (*e*.*g*. 2-AP^41^, A^T^^42^, and BPP^9^), and is commonly attributed to the electron donating properties of guanine^43^. In contrast, thymine has the lowest propensity of the four natural DNA-bases to donate electrons^43^. This suggests that electron transfer from guanine plays an important role in the quenching of bT inside DNA.

Overall, the brightness of bT is quenched when transitioning from ssDNA to dsDNA (see Table S2). We therefore investigated the potential of bT as an internal DNA probe of DNA melting, by determining fluorescence melting curves on four samples (AA, AC, CT and TT). Samples were chosen to include one case where the difference in brightness arises only from the difference in molar absorptivity (AA) as well as three cases with various degrees of quenching upon duplex formation (AC, CT and TT). In all four cases there is a clear difference between the brightness at low and high temperature, see Figure S4. The melting temperature is on average 2.5 °C higher when determined by fluorescence compared to UV methods, for individual values see Table S3. The difference between the two methods could be due to that the UV-measurements shows the full melting whereas the fluorescence data indicate local melting around the bT probe.

The photophysical features of bT follow similar trends as most of the thymine FBAs that have been characterized inside nucleic acids. Only a few thymine analogues have been characterized in nucleic acids, and most of those have been studied in one or just a few sequence contexts. In these studies, moderate to significant fluorescent quenching has been reported for 5-(furan-2-yl)-2′-deoxyuridine^25^, xT^27^, 1PydU and 2PydU^28^ inside DNA. One notable exception to this trend is ^DMA^T, where the quantum yield increases upon incorporation into duplex DNA^26^. Overall continued development of novel fluorescent thymine analogues and more thorough characterization of previously reported ones in various nucleic acid contexts will be important for their future application in chemistry, biology and nanotechnology.

## Conclusion

In summary, we have described a reliable synthetic route to the deoxyribose version of bicyclic thymine (bT), one that we foresee will prove to be a useful and general pathway for a range of bT-derivatives. Furthermore, we have demonstrated that bT acts as an excellent analogue of thymine and exhibits the same base-pairing characteristics as native thymine. bT is moderately fluorescent (ε·Φ_F_ = 790 M^−1^ cm^−1^) in water, and, like most other thymine analogues, experiences significant quenching upon incorporation into DNA. A thorough investigation of nearest neighbour dependency of the quantum yield of a fluorescent thymine analogue is evaluated here for the first time. It reveals that bT is brightest when flanked by thymines, and most quenched when flanked by guanines, suggesting that electron transfer is the principal quenching mechanism. Overall, bT appears to be an excellent thymine analogue, and we envision that the bT scaffold, like qA was for our bright adenine analogues qAN1 and pA, will serve as a strong starting point for the development of bright thymine analogues that retain the essential ability to form stable duplexes and to base-pair specifically with adenine.

## Methods

### Materials and instruments

All reactions were performed in flame-dried or oven-dried glassware under a nitrogen atmosphere unless otherwise noted. Reagents were purchased from various chemical vendors and either used as received or purified according to standard techniques. All solvents used for reactions were HPLC-grade and purchased dry. Microwave reactions were performed with a Biotage Initiator using single mode microwave irradiation with temperature and pressure control and with fixed hold time on. Reactions were monitored by TLC on silica gel plates analyzed under UV (254 nm), and by UPLC-MS (ESI/UV), using a Waters Acquity system equipped with either an Acquity UPLC HSS C18 column (1.8 µm, length 50 mm, ID 2.1 mm) running a gradient of water-MeCN (95:5) to water-MeCN (5:95), with the water eluent containing 1% formic acid (pH 3) or an Acquity UPLC BEH C18 column (1.7μm, length 50 mm, ID 2.1 mm) running a gradient of water-MeCN (95:5) to water-MeCN (5:95), with the water eluent containing 1% ammonium hydroxide (pH 10). Semi-automated flash column chromatography was performed on a Biotage HPFC SP4 Flash Purification System using pre-packed silica columns. HPLC purification was performed with ammonia as modifier on a preparative HPLC system with an Xbridge C18 column (10 µm, 250 × 50 mm). ^1^H and ^13^C NMR spectra were recorded at 300 K on a Bruker 500 MHz system equipped with a CryoProbe. All chemical shifts are recorded in ppm and were calibrated relative to the deuterated solvent: CD_2_Cl_2_ (5.32 ppm for ^1^H and 54.00 ppm for ^13^C) or DMSO-*d*_6_ (2.50 ppm for ^1^H and 39.52 ppm for ^13^C). 2D-NMR spectra (COSY) were used for detection of peaks overlapping with the deuterated solvent. ^31^P NMR spectra were recorded on a Bruker AVIII400 nanobay instrument (162 MHz), and referenced to external 85% orthophosphoric acid (0.00 ppm). LRMS analysis was performed on a Xevo G2-XS QT of Quadrupole Time-of-Flight mass spectrometer with a Waters Acquity CSH C18 column (1.7 µm, length 100 mm, ID 2.1 mm) running a gradient of 1–95% MeCN in water containing 0.1% formic acid.

### Incorporation of bT into DNA-oligonucleotides and their purification

The oligonucleotide synthesis was carried out on an Applied Biosystems 394 automated DNA/RNA synthesiser using a standard 1.0 μmole phosphoramidite cycle of acid-catalyzed detritylation, coupling, capping and iodine oxidation. All β-cyanoethyl phosphoramidite monomers were dissolved in anhydrous acetonitrile to a concentration of 0.1 M immediately prior to use. The coupling time for normal A, G, C, and T monomers was 60 s and this was extended to 840 s for the bT monomer. Stepwise coupling efficiencies and overall yields were determined by automated trityl cation conductivity monitoring and in all cases were >98.0%. Cleavage of oligonucleotides from the solid support and deprotection were achieved by exposure to concentrated aqueous ammonia for 60 min at room temperature followed by heating in a sealed tube for 5 h at 55 °C. Purification of oligonucleotides was carried out by reversed-phase HPLC on a Gilson system using a Brownlee Aquapore column (C8, 8 mm × 250 mm, 300 Å pore) with a gradient of MeCN in aqueous triethylammonium bicarbonate (TEAB) increasing from 0% to 50% buffer B over 30 min with a flow rate of 4 mL/min (buffer A: 0.1 M TEAB, pH 7.0, buffer B: 0.1 M TEAB, pH 7.0 with 50% acetonitrile). Elution of oligonucleotides was monitored by ultraviolet absorption at 295 or 300 nm. After HPLC purification, oligonucleotides were freeze-dried then dissolved in water without the need for desalting. All oligonucleotides were characterized by electrospray mass spectrometry using a Bruker micrOTOF II focus ESI-TOF MS instrument in ESI-mode. Data were processed using MaxEnt.

### Molar absorptivity of the bT monomer

The molar absorptivity of the bT nucleoside in water and EtOH was determined with bT samples of known concentration in water (1, 2 and 4 μM) and EtOH (2 and 6 µM). All samples were prepared from a 3 mM stock of bT in EtOH (the final bT samples in water contained 0.16%, 0.32% and 0.64% EtOH, respectively). Absorption was measured between 200 and 500 nm using a Cary 5000 (Varian Technologies) with the spectral bandwidth set to 1 nm and at a scan rate of 200 nm min^−1^. Using the Beer-Lambert law, the molar absorptivity of bT at the maximum of the lowest energy transition and at 260 nm was determined for both solvents.

### Preparation of oligonucleotide samples

Sodium phosphate buffer (12.5 mM phosphate, 150 mM Na^+^, pH 7.4) was used for all measurements unless otherwise stated. Before hybridization, absorption spectra between 230 and 500 nm were recorded on a Cary 5000 (Varian Technologies) for each single strand. The absorption at 260 nm was used for calculating the concentration, where the oligonucleotide molar absorptivity at 260 nm was taken as the linear combination of the molar absorptivity of the individual bases at this wavelength, multiplied by 0.9 to account for the effect of base stacking. The values used for the molar absorptivity of each base at 260 nm are: ε(T) = 9300 M^−1^ cm^−1^, ε(C) = 7400 M^−1^ cm^−1^, ε(G) = 11800 M^−1^ cm^−1^, ε(A) = 15300 M^−1^ cm^−1^ and ε(bT) = 4500 M^−1^ cm^−1^. Hybridization was achieved by mixing each bT-modified strand with 15% excess of its complementary strand (to assure full hybridization of the modified strands) at room temperature, followed by heating to 95 °C and after 10 minutes at 95 °C cooling to 5 °C over a period of 12 hours. By measuring absorption on the single stranded DNA and hybridized duplexes (assuming the concentration is given by the absorption at 260 nm using the molar absorptivities of the DNA bases as stated above) the molar absorptivity of bT in ss- and dsDNA at the maximum of the lowest energy transition were determined using the Beer-Lambert law.

### DNA UV-melting and circular dichroism (CD)

DNA melting curves were recorded on a Cary 4000 (Varian Technologies) with a programmable multi-cell temperature block, by heating from 10 °C to 80 °C with a rate of 0.5 °C/min and subsequent cooling to 10 °C at the same rate. The absorption at 260 nm was recorded every 0.5 °C for two cycles. The duplex concentration was 3 μM in all measurements. The melting temperatures were calculated as the maximum of the first derivative of all four UV-melting/annealing curves after FFT-filtered smoothing. Circular dichroism (CD) spectra were recorded on a Chirascan CD spectrometer (Applied Photophysics) scanning between 200–450 nm, using a bandwidth of 1 nm for both excitation and emission, an integration time of 0.5 s and four repetitions. The duplex concentration was 6 μM in all measurements, and all spectra were corrected for background contribution.

### Fluorescence measurements

Steady-state emission spectra were recorded on a SPEX Fluorolog 3 (JY Horiba) using an excitation wavelength of 325 nm. The emission was recorded between 330 and 640 nm at a scan rate of 600 nm min^−1^, with the excitation and emission monochromator slit widths set to 1.5 and 6 nm, respectively. Quantum yields were determined using quinine sulphate (Φ_F_* = *54.6%) in 0.5 M H_2_SO_4_ as reference, using the same settings as above, but recording the emission between 330–700 nm. The quantum yield, Φ_F_, is calculated as:

$${{\rm{\Phi }}}_{{\rm{F}}}={{\rm{\Phi }}}_{{\rm{F}},{\rm{ref}}}\frac{{\eta }^{2}}{{\eta }_{{\rm{ref}}}^{2}}\frac{I}{{I}_{{\rm{ref}}}}\frac{{A}_{{\rm{ref}}}}{A}$$

where *η* is the refractive index of the solvent, *I* is the integrated fluorescence intensity and *A* is the absorbance at the excitation wavelength. All measurements were performed at least twice, using the monomer sample concentrations listed above, and a sample concentration of 6 µM both for ssDNA and dsDNA.

### Fluorescence melting

Fluorescence melting curves were recorded on a Cary Eclipse (Varian Technologies) with a programmable multi-cell temperature block, by heating from 20 °C to 85 °C with a rate of 0.5 °C/min and subsequent cooling to 20 °C at the same rate using an excitation wavelength of 325 nm. The emission at 380 nm was recorded every 0.5 °C for two cycles with the excitation and emission monochromator slit widths both set to 5 nm. The duplex concentration was 6 μM in all measurements. The melting temperatures were calculated as the maximum of the first derivative of all four UV-melting/annealing curves after FFT-filtered smoothing.

## Electronic supplementary material

Supporting Information

## References

[1] Yang SK, Shi X, Park S, Ha T, Zimmerman SC (2013). A dendritic single-molecule fluorescent probe that is monovalent, photostable, and minimally blinking. Nat. Chem..

[2] Juskowiak B (2011). Nucleic acid-based fluorescent probes and their analytical potential. Anal. Bioanal. Chem..

[3] Preus S, Wilhelmsson LM (2012). Advances in Quantitative FRET-Based Methods for Studying Nucleic Acids. Chem Bio Chem.

[4] Wilhelmsson, L. M. & Tor, Y. *Fluorescent Analogs of Biomolecular Building Blocks: Design and Applications*. (John Wiley & Sons, 2016). 10.1021/cr900301e PMC2868948 20205430

[5] Wiegant, J., Brouwer, A. K., Tanke, H. J. & Dirks, R. W. *Visualizing Nucleic Acids in Living Cells by Fluorescence In Vivo Hybridization*. Vol. 659 239–246 (Humana Press, 2010). 10.1007/978-1-60761-789-1_17 20809316

[6] Xu W, Chan KM, Kool ET (2017). Fluorescent nucleobases as tools for studying DNA and RNA. Nat. Chem..

[7] Sinkeldam RW, Greco NJ, Tor Y (2010). Fluorescent analogs of biomolecular building blocks: Design, properties, and applications. Chem. Rev..

[8] Wilhelmsson LM (2010). Fluorescent nucleic acid base analogues. Q. Rev. Biophys..

[9] Okamoto A, Saito Y, Saito I (2005). Design of base-discriminating fluorescent nucleosides. J. Photochem. Photobiol. C.

[10] Ward DC, Reich E, Stryer L (1969). Fluorescence Studies of Nucleotides and Polynucleotides: I. Formycin, 2-aminopurine riboside, 2,6-diaminopurine riboside, and their derivatives. J. Biol. Chem..

[11] Eldrup AB (2001). 1,8-Naphthyridin-2(1*H*)-ones − Novel Bicyclic and Tricyclic Analogues of Thymine in Peptide Nucleic Acids (PNAs). Eur. J. Org. Chem..

[12] Eldrup AB, Christensen C, Haaima G, Nielsen PE (2002). Substituted 1,8-Naphthyridin-2(1*H*)-ones Are Superior to Thymine in the Recognition of Adenine in Duplex as Well as Triplex Structures. J. Am. Chem. Soc..

[13] Sandin P, Lincoln P, Brown T, Wilhelmsson LM (2007). Synthesis and oligonucleotide incorporation of fluorescent cytosine analogue tC: a promising nucleic acid probe. Nat. Protocols.

[14] Sandin P (2008). Characterization and use of an unprecedentedly bright and structurally non-perturbing fluorescent DNA base analogue. Nucleic Acids Res..

[15] Börjesson K (2009). Nucleic Acid Base Analog FRET-Pair Facilitating Detailed Structural Measurements in Nucleic Acid Containing Systems. J. Am. Chem. Soc..

[16] Dierckx A (2012). Quadracyclic Adenine: A Non-Perturbing Fluorescent Adenine Analogue. Chem. Eur. J..

[17] Dumat B (2015). Second-Generation Fluorescent Quadracyclic Adenine Analogues: Environment-Responsive Probes with Enhanced Brightness. Chem. Eur. J..

[18] Wranne MS (2017). Toward Complete Sequence Flexibility of Nucleic Acid Base Analogue FRET. J. Am. Chem. Soc..

[19] Bood, M. *et al*. Pentacyclic adenine: a versatile and exceptionally bright fluorescent DNA base analogue. *Chem*, *Sci*. **9**, 3494–3502 (2018). 10.1039/c7sc05448c PMC5934695 29780479

[20] Danishefsky SJ, Bilodeau MT (1996). Glycals in Organic Synthesis: The Evolution of Comprehensive Strategies for the Assembly of Oligosaccharides and Glycoconjugates of Biological Consequence. Angew. Chem. Int. Ed..

[21] Temburnikar K, Zhang Z, Seley-Radtke K (2012). Modified Synthesis of 3′-OTBDPS-Protected Furanoid Glycal. Nucleosides Nucleotides Nucleic Acids.

[22] Walker JA, Chen JJ, Wise DS, Townsend LB (1996). A Facile, Multigram Synthesis of Ribofuranoid Glycals. J. Org. Chem..

[23] Liverton, N. J. *et al*. Preparation of macrocyclic peptides as HCV NS3 protease inhibitors. PCT Int. Appl. WO 2008057209 (2008).

[24] Klapars A, Buchwald SL (2002). Copper-Catalyzed Halogen Exchange in Aryl Halides:  An Aromatic Finkelstein Reaction. J. Am. Chem. Soc..

[25] Greco NJ, Tor Y (2005). Simple Fluorescent Pyrimidine Analogues Detect the Presence of DNA Abasic Sites. J. Am. Chem. Soc..

[26] Mata G, Schmidt OP, Luedtke NW (2016). A fluorescent surrogate of thymidine in duplex DNA. Chem. Commun..

[27] Liu H, Gao J, Maynard L, Saito YD, Kool ET (2004). Toward a New Genetic System with Expanded Dimensions:  Size-Expanded Analogues of Deoxyadenosine and Thymidine. J. Am. Chem. Soc..

[28] Wanninger-Weiß C, Wagenknecht H-A (2008). Synthesis of 5-(2-Pyrenyl)-2′-deoxyuridine as a DNA Modification for Electron-Transfer Studies: The Critical Role of the Position of the Chromophore Attachment. Eur. J. Org. Chem..

[29] Shin D, Sinkeldam RW, Tor Y (2011). Emissive RNA alphabet. J. Am. Chem. Soc..

[30] Barthes NPF (2015). Development of environmentally sensitive fluorescent and dual emissive deoxyuridine analogues. RSC Adv..

[31] Godde F, Aupeix K, Moreau S, Toulmé J-J (1998). A Fluorescent Base Analog for Probing Triple Helix Formation. Antisense Nucleic Acid Drug Dev..

[32] Kypr J, Kejnovská I, Renciuk D, Vorlicková M (2009). Circular dichroism and conformational polymorphism of DNA. Nucleic Acids Res..

[33] Engman KC (2004). DNA adopts normal B-form upon incorporation of highly fluorescent DNA base analogue tC: NMR structure and UV-Vis spectroscopy characterization. Nucleic Acids Res..

[34] Johnson NP, Baase WA, von Hippel PH (2004). Low-energy circular dichroism of 2-aminopurine dinucleotide as a probe of local conformation of DNA and RNA. Proc. Natl. Acad. Sci. USA.

[35] Füchtbauer AF (2017). Fluorescent RNA cytosine analogue – an internal probe for detailed structure and dynamics investigations. Sci. Rep..

[36] Lee AHF, Kool ET (2005). Novel Benzopyrimidines as Widened Analogues of DNA Bases. J. Org. Chem..

[37] Allawi HT, SantaLucia J (1998). Thermodynamics of internal C·T mismatches in DNA. Nucleic Acids Res..

[38] Gaffney BL, Jones RA (1989). Thermodynamic comparison of the base pairs formed by the carcinogenic lesion *O*^6^-methylguanine with reference both to Watson-Crick pairs and to mismatched pairs. Biochemistry.

[39] Aboul-ela F, Koh D, Tinoco I, Martin FH (1985). Base-base mismatches. Thermodynamics of double helix formation for dCA_3_XA_3_G + dCT_3_YT_3_G (X, Y = A,C,G,T). Nucleic Acids Res..

[40] Cantor, C. R. & Schimmel, P. R. *Biophysical Chemistry - Part II: Techniques for the Study of Biological Structure and Function*. 403 (Freeman, W.H. and Company, 2001).

[41] Somsen OJG, Hoek VA, Amerongen VH (2005). Fluorescence quenching of 2-aminopurine in dinucleotides. Chem. Phys. Lett..

[42] Dierckx A (2011). Characterization of photophysical and base-mimicking properties of a novel fluorescent adenine analogue in DNA. Nucleic Acids Res..

[43] Seidel CAM, Schulz A, Sauer MHM (1996). Nucleobase-Specific Quenching of Fluorescent Dyes. 1. Nucleobase One-Electron Redox Potentials and Their Correlation with Static and Dynamic Quenching Efficiencies. J. Phys. Chem..

