# Supplementary material for: Synthesis, oligonucleotide incorporation and fluorescence properties in DNA of a bicyclic thymine analogue
Source: Sci Rep. 2018 Sep 18;8:13970. doi: 10.1038/s41598-018-31897-2 (PMC6143597; doi:10.1038/s41598-018-31897-2)
Supplement: Supplementary file 1 — Supporting Information [file 41598_2018_31897_MOESM1_ESM.docx]

Supporting Information for:

**Synthesis, oligonucleotide incorporation and fluorescence properties in DNA of a bicyclic thymine analogue**

Christopher P. Lawson, Anders F. Füchtbauer, Moa S. Wranne, Tristan Giraud, Thomas Floyd, Blaise Dumat, Nicolai K. Andersen, Afaf El-Sagheer, Tom Brown, Henrik Gradén, L. Marcus Wilhelmsson, Morten Grøtli

**Table of Contents**

[Synthesis and characterization of bicyclic thymine analogues 2](#_Toc519028811)

[Spectral Data 6](#_Toc519028812)

[Additional figures and tables 14](#_Toc519028813)

[Figure S1. 14](#_Toc519028814)

[Figure S2. 15](#_Toc519028815)

[Figure S3 16](#_Toc519028816)

[Figure S4. 16](#_Toc519028817)

[Table S1. 17](#_Toc519028818)

[Table S2. 17](#_Toc519028819)

[Table S3. 18](#_Toc519028820)

[References 18](#_Toc519028821)

# Synthesis and characterization of bicyclic thymine analogues

**1-(5'-*O*-(*tert*-Butyldimethylsilyl)-2'-deoxy-β-d-ribofuranosyl)-5-methylpyrimidine-2,4(1*H*,3*H*)-dione (11)**

Thymidine (1 g, 4.13 mmol), imidazole (0.337 g, 4.95 mmol), and TBDMS-Cl (0.653 g, 4.33 mmol) were added to a 20 mL microwave vial, sealed, and flushed with nitrogen. DMF (5 mL) was then added and the resulting reaction mixture was stirred at RT overnight. The reaction was quenched with water and extracted with EtOAc (3 × 50 mL). The combined organic phases were washed with satd. aq. NaHCO_3_ and brine, and dried over MgSO_4_. Purification by automated flash column chromatography (MeOH in DCM; 0-10%) afforded the title compound (1.23 g, 3.44 mmol, 83%) as a clear oil. ^1^H NMR (500 MHz, DMSO-*d*_6_) δ 11.31 (s, 1H), 7.47 (d, *J* = 1.3 Hz, 1H), 6.16 (dd, *J* = 7.7, 6.2 Hz, 1H), 5.26 (d, *J* = 4.2 Hz, 1H), 4.23–4.16 (m, 1H), 3.76–3.82 (m, 2H), 3.72 (dd, *J* = 11.2, 3.7 Hz, 1H), 2.12–2.02 (m, 2H), 1.77 (d, *J* = 1.2 Hz, 3H), 0.88 (s, 9H), 0.08 (s, 3H), 0.07 (s, 3H); ^13^C NMR (126 MHz, DMSO-*d*_6_) δ 163.6, 150.3, 135.5, 109.4, 86.8, 83.8, 70.5, 63.3, 39.4 (overlaps with DMSO), 25.8, 18.0, 12.2, -5.42, -5.44; LRMS (ES^+^): *m/z* (%), 357 (100) [M+H]^+^.

**1-(5'-*O*-(*tert*-Butyldimethylsilyl)-4'-*O-*(*tert*-butyldiphenylsilyl)-2'-deoxy-β-d-ribofuranosyl)-5-methylpyrimidine-2,4(1*H*,3*H*)-dione (8)**

Compound **11** (1.228 g, 3.44 mmol) and imidazole (0.352 g, 5.17 mmol) were added to a 20 mL microwave vial, sealed, and flushed with nitrogen. DMF (10 mL) was then added, followed by drop-wise addition of TBDPS-Cl (0.929 mL, 3.62 mmol), and the resulting reaction was stirred at 60 °C overnight. The reaction was quenched with water and extracted with EtOAc (3 × 50 mL). The combined organic layers were washed with satd. aq. NaHCO_3_ and brine, dried over MgSO_4_, and concentrated under reduced pressure. Purification by automated flash column chromatography (EtOAc in pentane: 0-20%) afforded the title compound in quantitative yield as a white foam (2.04 g, 3.43 mmol, 100%). ^1^H NMR (500 MHz, DMSO-*d*_6_) δ 11.34 (s, 1H), 7.63–7.58 (m, 4H), 7.51–7.47 (m, 2H), 7.47–7.42 (m, 4H), 7.33 (d, *J* = 1.2 Hz, 1H), 6.29 (dd, *J* = 8.7, 5.6 Hz, 1H), 4.34–4.27 (m, 1H), 3.98–3.92 (m, 1H), 3.55 (dd, *J* = 11.4, 3.4 Hz, 1H), 3.29 (dd, *J* = 11.4, 3.4 Hz, 1H), 2.14 (ddd, *J* = 13.2, 5.6, 1.8 Hz, 1H), 1.94 (ddd, *J* = 13.5, 8.8, 5.6 Hz, 1H), 1.72 (d, *J* = 1.0 Hz, 3H), 1.04 (s, 9H), 0.74 (s, 9H), -0.09 (s, 3H), -0.12 (s, 3H); ^13^C NMR (126 MHz, DMSO-*d*_6_) δ 163.5, 150.3, 135.2, 135.1, 132.70, 132.64, 130.1, 128.00, 127.98, 109.5, 86.9, 83.9, 73.8, 62.8, 39.5 (overlaps with DMSO), 26.7, 25.6, 18.6, 17.8, 12.2, -5.6, -5.8; LRMS (ES^+^): *m/z* (%), 595 (100) [M+H]^+^.

**1-(4'-*O-*(*tert*-Butyldiphenylsilyl)-2'-deoxy-β-d-ribofuranosyl)-5-methylpyrimidine-2,4(1*H*,3*H*)-dione (9)**

A solution of **8** (6.00 g, 10.09 mmol) in CH_2_Cl_2_ (20 mL) in a round bottom flask was cooled to 0 °C in an ice bath. A solution of TFA in water (TFA:H_2_O 10:1, 12 mL) was added and the resultant mixture was stirred vigorously at 0 °C for 4 h. The reaction was quenched with satd. aq. NaHCO_3_ and extracted with DCM (3 × 50 mL). The combined organic phases were dried over MgSO_4_ and concentrated under reduced pressure. Purification by automated flash column chromatography (EtOAc in pentane: 30-100%) afforded the title compound as a white foam (4.48 g, 9.32 mmol, 92%).^1^H NMR (500 MHz, CD_2_Cl_2_) δ 9.75 (s, 1H), 7.73–7.64 (m, 4H), 7.50–7.39 (m, 6H), 7.35 (d, *J* = 1.2 Hz, 1H), 6.28 (dd, *J* = 7.7, 6.1 Hz, 1H), 4.47 (dt, *J* = 6.1, 3.0 Hz, 1H), 4.01 (q, *J* = 2.9 Hz, 1H), 3.62 (dd, *J* = 12.0, 2.7 Hz, 1H), 3.28 (dd, *J* = 12.0, 3.3 Hz, 1H), 2.69 (s, 1H), 2.30 (ddd, *J* = 13.4, 6.1, 3.0 Hz, 1H), 2.12 (ddd, *J* = 13.7, 7.8, 6.2 Hz, 1H), 1.82 (d, *J* = 1.3 Hz, 3H), 1.11 (s, 9H).; ^13^C NMR (126 MHz, CD_2_Cl_2_) δ 164.9, 151.2, 137.4, 136.32, 136.29, 133.87, 133.78, 130.59, 130.55, 128.40, 128.39, 111.3, 88.3, 86.9, 73.7, 62.5, 40.8, 27.2, 19.4, 12.7.; LRMS (ES^+^): *m/z* (%), 481 (100) [M+H]^+^, 961 (60) [2M+H]^+^; LRMS (ES^-^): *m/z* (%), 959 (40) [2M-H]^–^.

**(2*R*,3*S*)-3-((*tert*-Butyldiphenylsilyl)oxy)-2,3-dihydrofuran-2-yl)methanol (3)**

Compound **9** (2.30 g, 4.79 mmol) and ammonium sulphate (0.206 g, 1.56 mmol) were added to a microwave vial, sealed, and flushed with nitrogen. HMDS (30 mL, 143 mmol) was then added, and the resulting suspension was heated at 80 °C until a clear solution was obtained. The reaction was cooled to room temperature and TMS-Cl (0.798 mL, 6.24 mmol) was added, resulting in the formation of a white precipitate. The mixture was then heated at reflux for a further 4 h, cooled, and concentrated under reduced pressure. The crude mixture was partitioned between DCM and H_2_O (100 mL, 1:1). The organic layer was washed with satd. aq. NaHCO_3_ and brine, dried over MgSO_4_, and concentrated under reduced pressure. Purification by automated flash column chromatography, (MeOH in DCM: 0-5%) afforded the title compound as a white foam (1.45 g, 4.09 mmol, 85%). ^1^H NMR (500 MHz, DMSO-*d_6_*) δ 7.65–7.60 (m, 4H), 7.49–7.40 (m, 6H), 6.61 (d, *J* = 2.1 Hz, 1H), 4.77–4.85 (m, 3H), 4.32 (td, *J* = 6.0, 2.4 Hz, 1H), 3.21 (dt, *J* = 11.7, 5.9 Hz, 1H), 3.11 (dt, *J* = 11.3, 5.5 Hz, 1H), 0.99 (s, 9H); ^13^C NMR (126 MHz, DMSO-*d_6_*) δ 149.7, 135.28, 135.23, 133.5, 133.3, 129.85, 127.83, 127.77, 102.8, 89.1, 76.7, 60.9, 26.7, 18.6; LRMS (ES^+^): *m/z* (%), 709 (100) [2M+H]^+^.

**3-Bromo-1,8-naphthyridin-2(1*H*)-one (4)**

A dry flask was charged with 2-oxo-1,2-dihydro-1,8-naphthyridine-3-carboxylic acid (**10**, 1.00 g, 5.26 mmol) and 4-dimethylaminopyridine (0.128 g, 1.05 mmol), sealed, and flushed with nitrogen. THF (10 mL) and pyridine (4.25 mL, 52.6 mmol) were added and the mixture was stirred for 5 min. Bromine (1.36 mL, 26.3 mmol) was added slowly (exotherm), followed by BBr_3_ (1.0 M, 1.052 mL, 1.05 mmol). The resulting solution was heated at 85 °C for 2 h, cooled, and the precipitate filtered and washed with THF to afford the title compound (1.10 g, 4.89 mmol, 93%), which was used in the next step without further purification. ^1^H NMR (500 MHz, DMSO-*d_6_*) δ 12.67 (s, 1H), 8.56 (dd, *J* = 4.8, 1.8 Hz, 1H), 8.54 (s, 1H), 8.12 (dd, *J* = 7.8, 1.8 Hz, 1H), 7.29 (dd, *J* = 7.8, 4.7 Hz, 1H); ^13^C NMR (126 MHz, DMSO-*d_6_*) δ 158.5, 150.8, 149.1, 140.5, 135.9, 118.7, 118.3, 114.5; HRMS (ESI^+^): *m/z* calcd for C_8_H_6_BrN_2_O^+^ [M+H]^+^: 224.9663, found: 224.9666.

**3-Iodo-1,8-naphthyridin-2(1*H*)-one (5)**

A vial was charged with **4** (0.214 g, 0.95 mmol), sodium iodide (0.285 g, 1.90 mmol), and copper (I) iodide (9.06 mg, 0.05 mmol), sealed, and flushed with nitrogen. Dioxane (2 mL), followed by *trans*-*N*,*N*'-dimethylcyclohexane-1,2-diamine (0.015 mL, 0.10 mmol), were added, and the resulting mixture was heated at 110 °C for 12 h, then cooled. Water was added, and the resulting precipitate was filtered off and washed with ethanol to afford the title compound (0.140 g, 0.51 mmol, 54%), which was used without further purification. ^1^H NMR (500 MHz, DMSO-*d_6_*) δ 12.22 (s, 1H), 8.74 (s, 1H), 8.57–8.52 (m, 1H), 8.12–8.06 (m, 1H), 7.25 (dd, *J* = 7.6, 4.4 Hz, 1H); ^13^C NMR (126 MHz, DMSO-*d_6_*) δ 159.7, 150.8, 149.8, 147.4, 135.4, 118.5, 115.4, 97.5; HRMS (ESI^+^): *m/z* calcd for C_8_H_6_IN_2_O^+^ [M+H]^+^: 272.9525, found: 272.9528.

**3-(2'-Deoxy-β-d-ribofuranosyl)-1,8-naphthyridin-2(1*H*)-one (1)**

Synthesised using a literature protocol.^1^ A mixture of diacetoxypalladium (0.050 g, 0.22 mmol) and triphenylarsine (0.096 mL, 0.44 mmol) in DMF (10 mL) was stirred at room temperature for 30 min. This suspension was added to a suspension of **5** (0.60 g, 2.21 mmol), **3** (0.860 g, 2.43 mmol), and tributylamine (0.579 mL, 2.43 mmol) in DMF (10 mL). The reaction mixture was heated to 60 °C for 16 h. Only 15% conversion was obtained; therefore, one more equivalent of catalyst (0.050 g, 0.22 mmol), triphenylarsine (0.096 mL, 0.44 mmol) and a further equivalent of glycal **3** (0.860 g, 2.43 mmol) were added, and the mixture was stirred for a further 16 h. The reaction mixture was cooled to 0 °C, and acetic acid (0.381 mL, 6.66 mmol) and TBAF (1.0 M in THF, 2.65 mL, 2.65 mmol) were added. After stirring for 45 min at 0 °C, the solvent was removed under reduced pressure. The residue was purified by silica gel column chromatography (7% MeOH in CH_2_Cl_2_) to give the 3'-keto derivative. This solid was dissolved in a mixture of acetonitrile and AcOH (40 mL, 1:1), and sodium triacetoxyborohydride (STAB, 0.608 g, 2.87 mmol) was added at 0 °C. The suspension was stirred for 1 h at 0 °C and quenched with acetone. The solvents were removed under reduced pressure, and the residue was purified by silica gel column chromatography (MeOH in CH_2_Cl_2_: 5-20%) followed by HPLC (pH 10) to afford the title compound (0.477 g, 1.82 mmol, 82%) as a white foam. ^1^H NMR (500 MHz, DMSO-*d_6_*) δ 12.18 (s, 1H), 8.47 (dd, *J* = 4.8, 1.8 Hz, 1H), 8.10 (dd, *J* = 7.8, 1.8 Hz, 1H), 7.97 (d, *J* = 1.3 Hz, 1H), 7.23 (dd, *J* = 7.7, 4.7 Hz, 1H), 5.11–5.02 (m, 2H), 4.79 (t, *J* = 5.5 Hz, 1H), 4.19–4.14 (m, 1H), 3.82 (td, *J* = 5.2, 2.4 Hz, 1H), 3.53 (dt, *J* = 9.4, 4.5 Hz, 1H), 3.45 (dt, *J* = 11.0, 4.6 Hz, 1H), 2.31 (ddd, *J* = 12.9, 5.9, 2.0 Hz, 1H), 1.65 (ddd, *J* = 12.7, 9.9, 5.7 Hz, 1H); ^13^C NMR (126 MHz, DMSO-*d_6_*) δ 161.8, 149.7, 148.9, 136.3, 136.1, 132.1, 118.4, 114.3, 87.3, 74.8, 72.2, 62.3, 41.2; HRMS (ESI^+^): *m/z* calcd for C_13_H_14_BrN_2_O^+^ [M+H]^+^: 263.1032, found: 263.1036.

**3-(2'-Deoxy-3'-*O*-[(2-cyanoethyl-*N*,*N*-diisopropyl)phosphoramidyl]-5'-*O*-(4,4'-dimethoxytrityl)-β-d-ribofuranosyl)-1,8-naphthyridin-2(1*H*)-one (2)**

A flask charged with **1** (0.350 g, 1.33 mmol) was sealed and flushed with nitrogen. Pyridine (10 mL) was added, and the resulting mixture was cooled to 0 °C. A solution of 4,4'-dimethoxytrityl chloride (0.520 g, 1.53 mmol) in pyridine (2 mL) was added slowly, and the resulting reaction was allowed to warm to RT, and was stirred at RT for a further 3 h. Pyridine was removed under reduced pressure (azeotrope with toluene). The crude product was then dissolved in DCM and washed with satd. aq. ammonium chloride, concentrated, and purified by HPLC (pH 10).

This material (293 mg, 0.519 mmol) was co-evaporated twice with dry DCM then dried overnight under vacuum. The dry compound was then dissolved in dry DCM (5 mL) and stirred over molecular sieves under an atmosphere of argon for 5 min. Then DIPEA (0.23 mL, 1.3 mmol) was added. 2-Cyanoethyl-*N*,*N*-diisopropylchloro-phosphoramidite (0.15 mL, 0.685 mmol) was added dropwise, and the reaction mixture was stirred at room temperature for 2 h and subsequently transferred under argon into a separating funnel containing degassed DCM (20 mL). The mixture was washed with degassed saturated aqueous KCl (20 mL) and the organic layer was separated, dried over sodium sulfate, filtered, and the solvent was removed in vacuo. The crude phosphoramidite product was dried under vacuum for 1 h, before it was precipitated by dissolving the dried product in anhydrous DCM (1 mL) and adding to it anhydrous hexane (100 mL) with vigorous stirring. After removing the solvent by decanting, the process was repeated three times, 0.368 g (93%) of the phosphoramidite monomer was obtained. ^1^H NMR (400 MHz, CD_2_Cl_2_) δ 11.39 – 10.81 (m, 1H), 8.65 – 8.56 (m, 1H), 8.00 (dd, *J* = 14.8, 1.4 Hz, 1H), 7.69 (ddd, *J* = 14.8, 7.8, 1.8 Hz, 1H), 7.54 – 7.44 (m, 2H), 7.42 – 7.33 (m, 4H), 7.31 – 7.26 (m, 2H), 7.25 – 7.20 (m, 1H), 7.16 (dt, *J* = 7.8, 4.9 Hz, 1H), 6.86 – 6.78 (m, 4H), 5.32 – 5.27 (m, 1H), 4.56 – 4.45 (m, 1H), 4.27 – 4.19 (m, 1H), 3.89 – 3.78 (m, 1H), 3.78 – 3.73 (m, 6H), 3.73 – 3.65 (m, 1H), 3.65 – 3.53 (m, 2H), 3.42 – 3.25 (m, 2H), 2.78 – 2.66 (m, 1H), 2.64 (t, *J* = 6.3 Hz, 1H), 2.47 (t, *J* = 6.4 Hz, 1H), 2.03 – 1.89 (m, 1H), 1.28 – 1.06 (m, 12H). ^31^P NMR (162 MHz, CD_2_Cl_2_) δ 148.2 (q, *J* = 9.0 Hz), 148.0 (q, *J* = 8.8 Hz).

## Spectral Data

**1-(5'-*O*-(*tert*-Butyldimethylsilyl)-2'-deoxy-β-d-ribofuranosyl)-5-methylpyrimidine-2,4(1*H*,3*H*)-dione (11)**


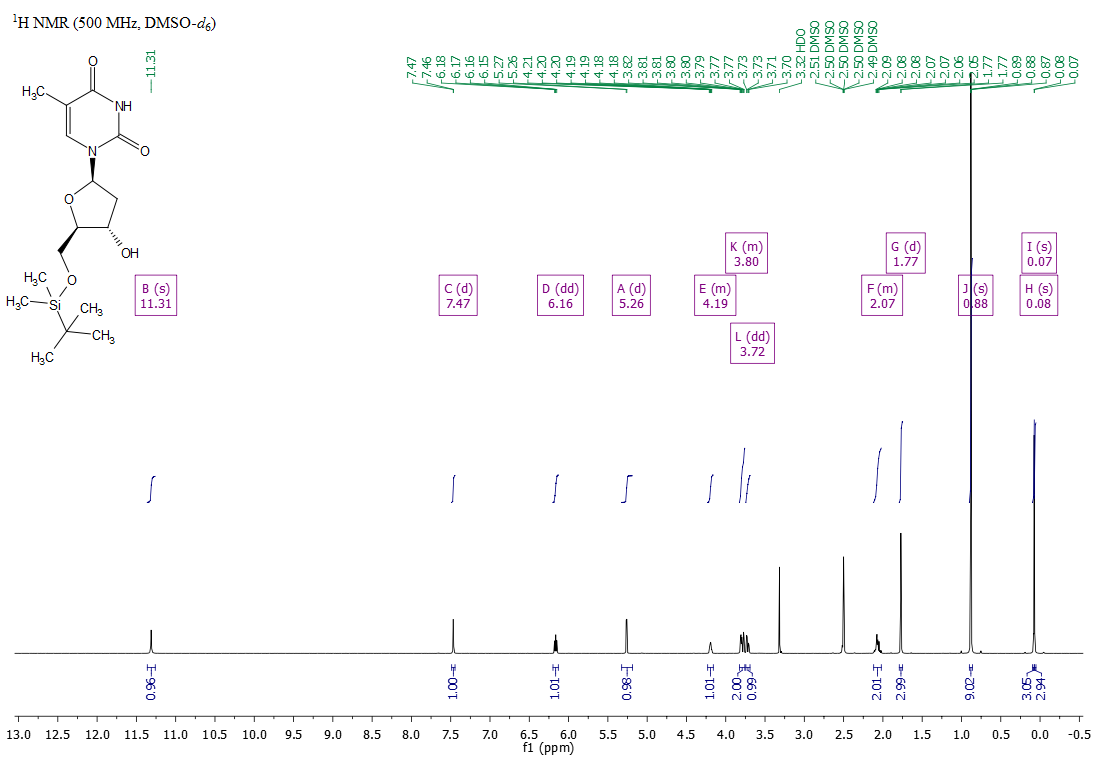


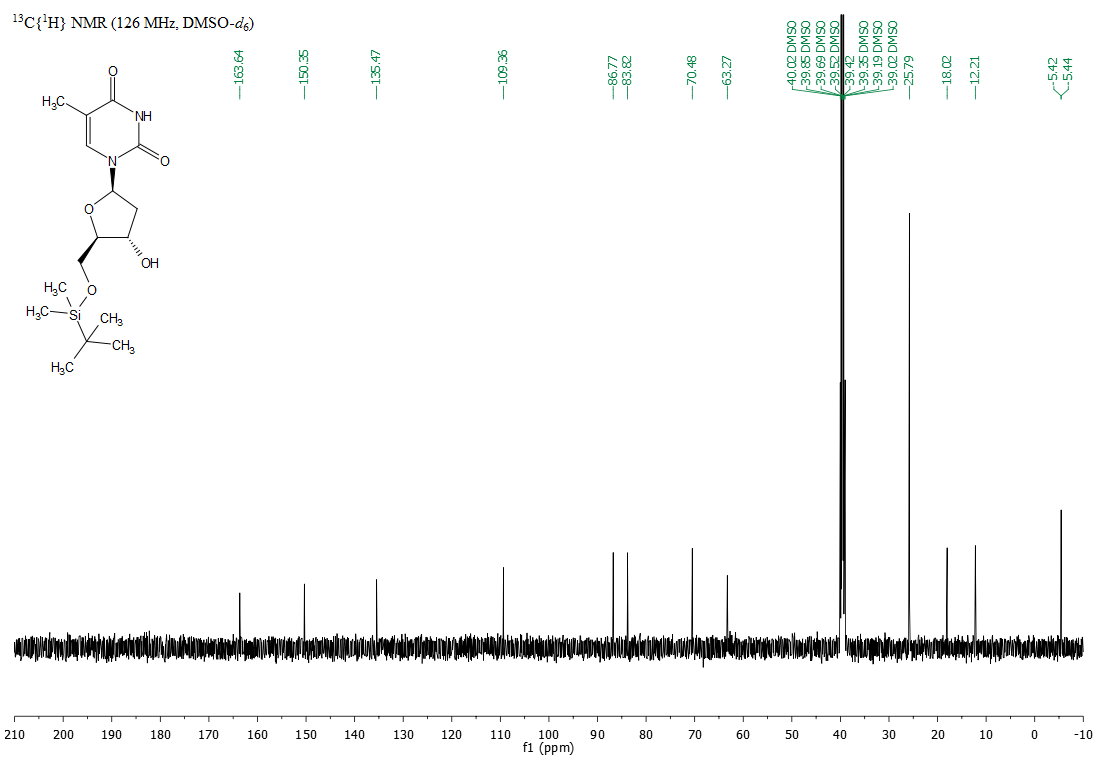


**1-(5'-*O*-(*tert*-Butyldimethylsilyl)-4'-*O-*(*tert*-butyldiphenylsilyl)-2'-deoxy-β-d-ribofuranosyl)-5-methylpyrimidine-2,4(1*H*,3*H*)-dione (8)**


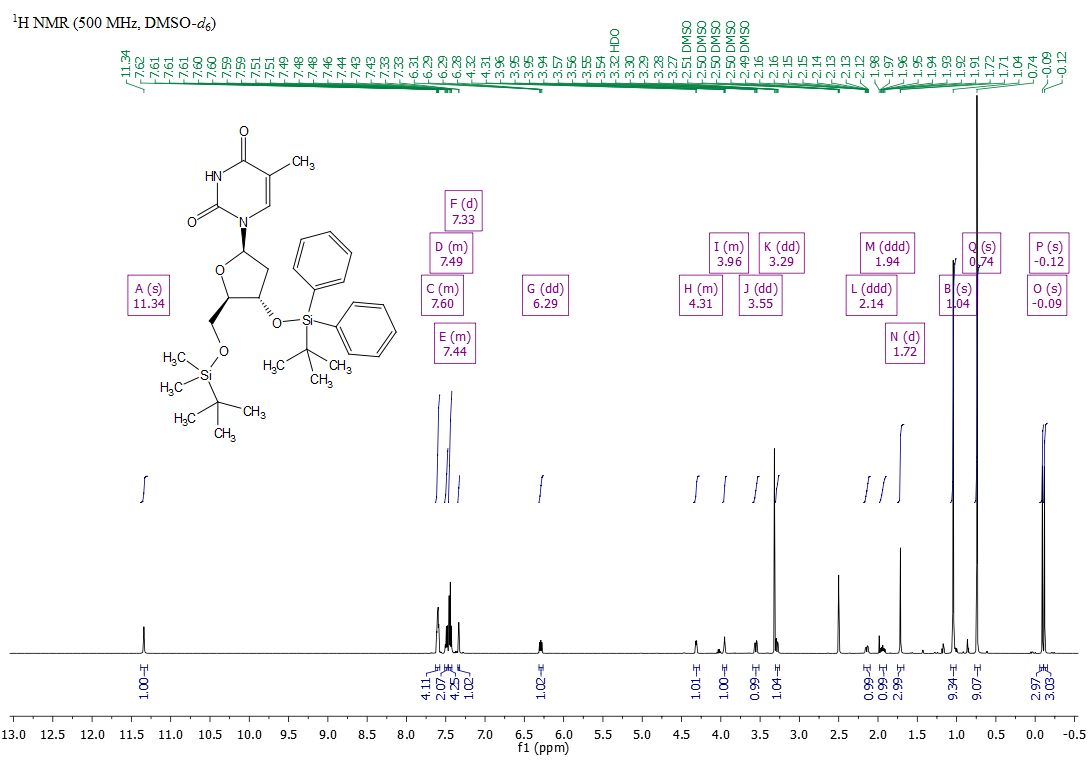


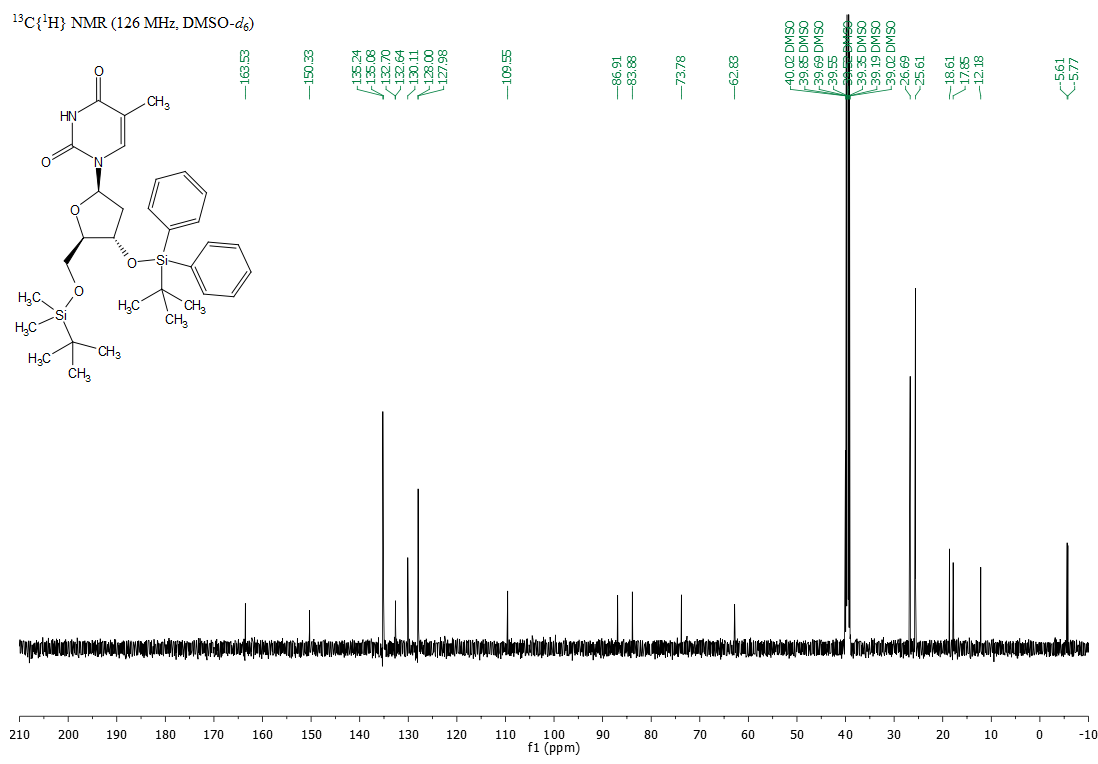


**1-(4'-*O-*(*tert*-Butyldiphenylsilyl)-2'-deoxy-β-d-ribofuranosyl)-5-methylpyrimidine-2,4(1*H*,3*H*)- dione (9)**


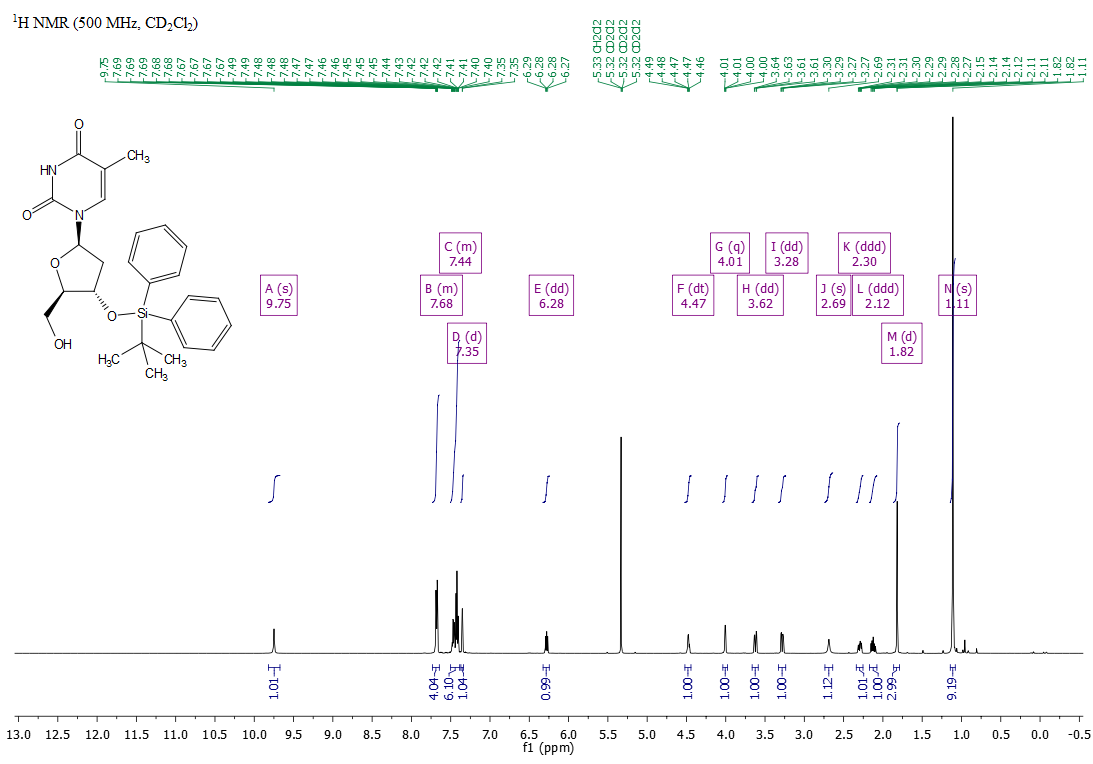


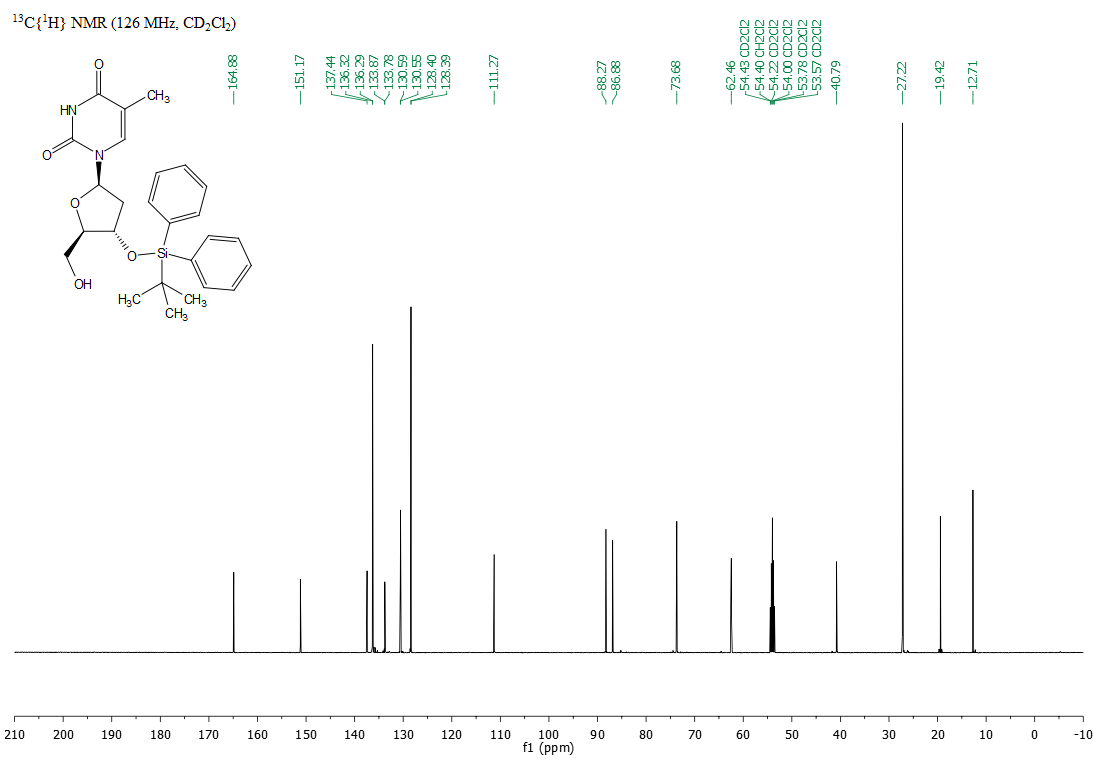


**(2*R*,3*S*)-3-((*tert*-Butyldiphenylsilyl)oxy)-2,3-dihydrofuran-2-yl)methanol (3)**


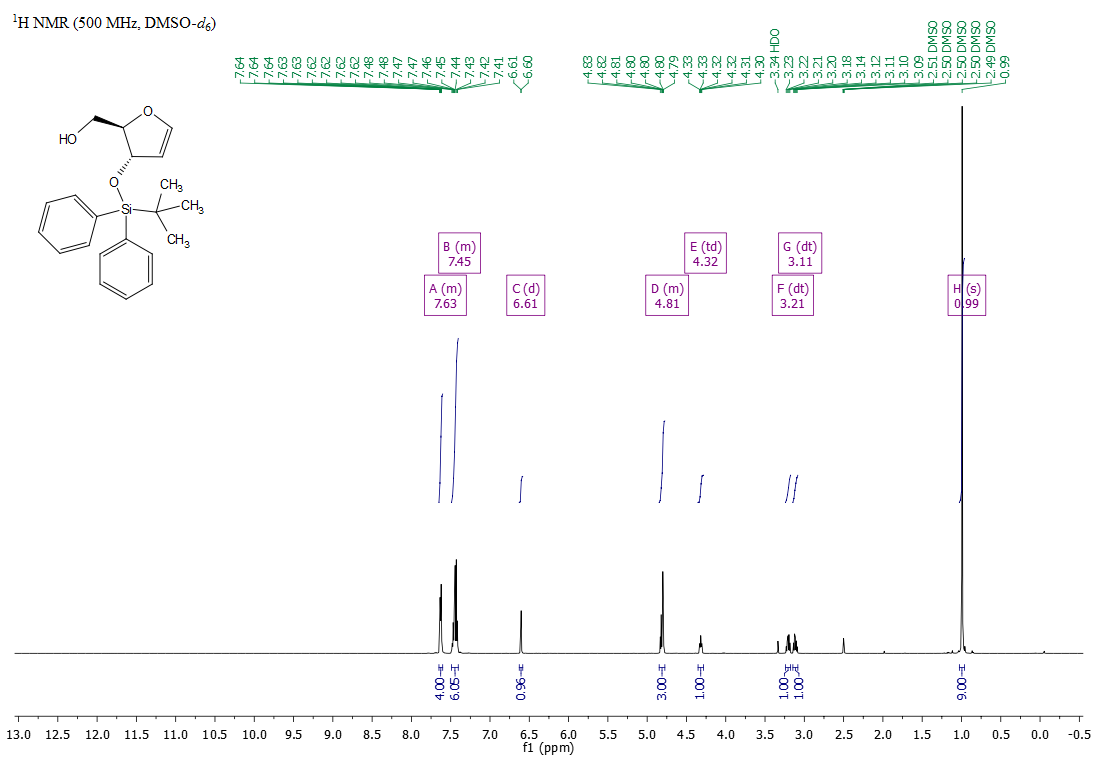


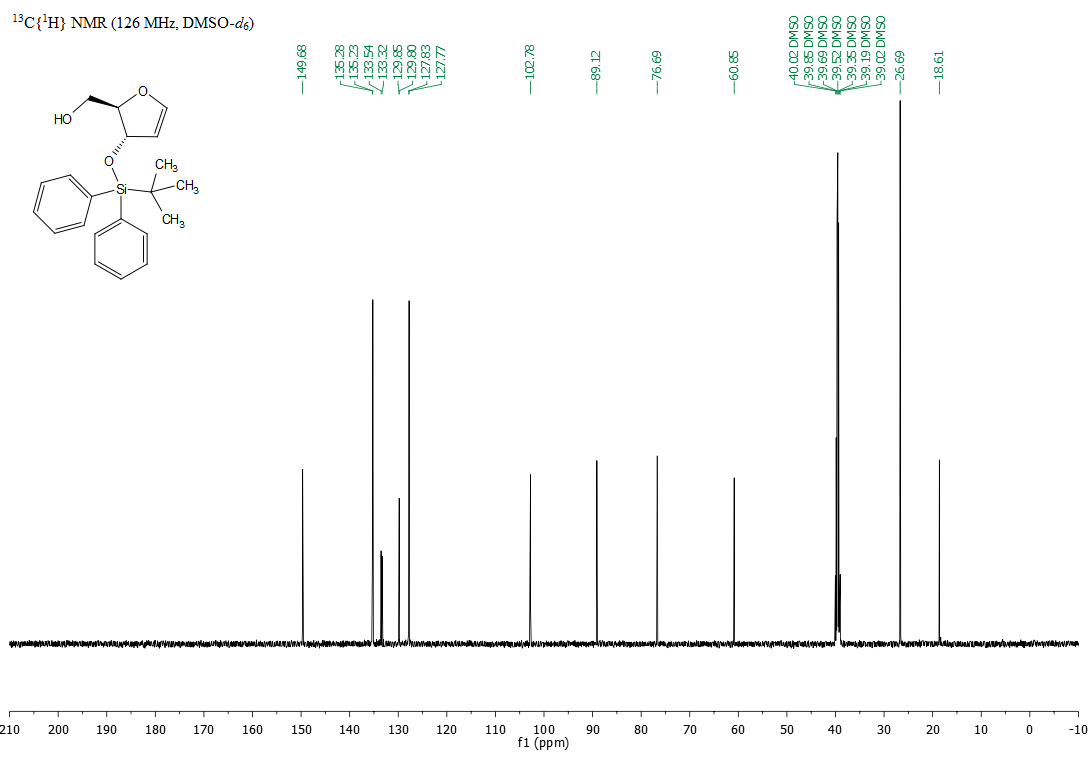


**3-Bromo-1,8-naphthyridin-2(1*H*)-one (4)**


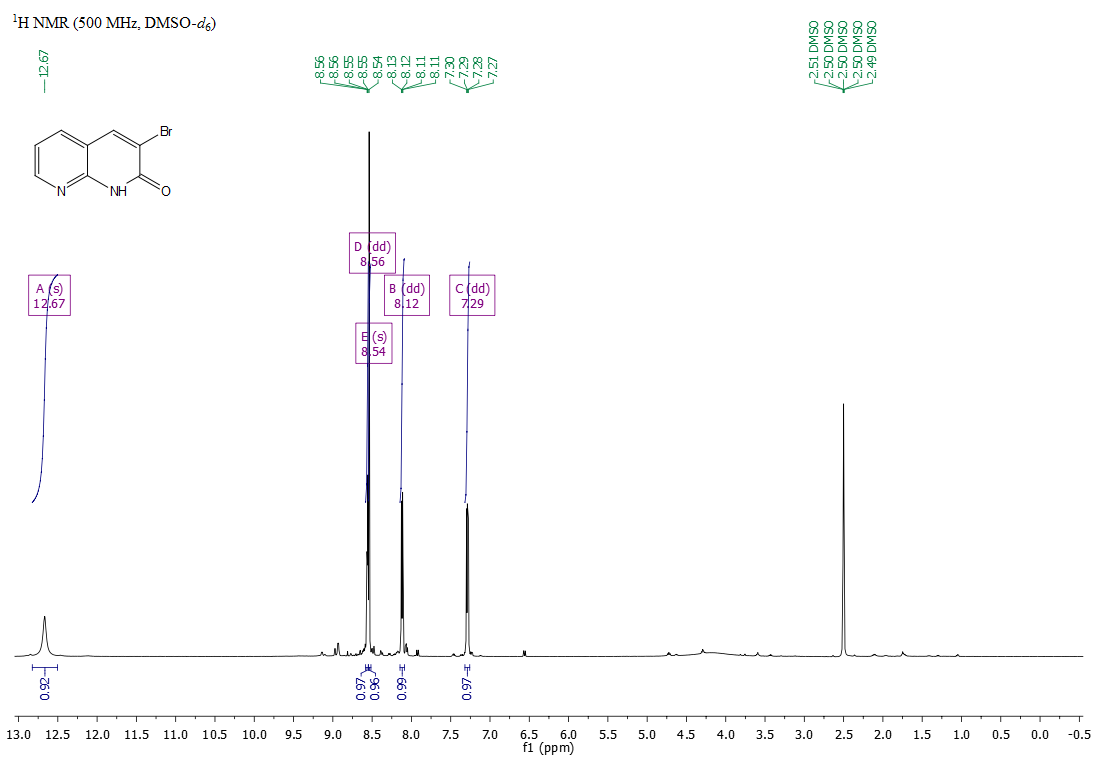


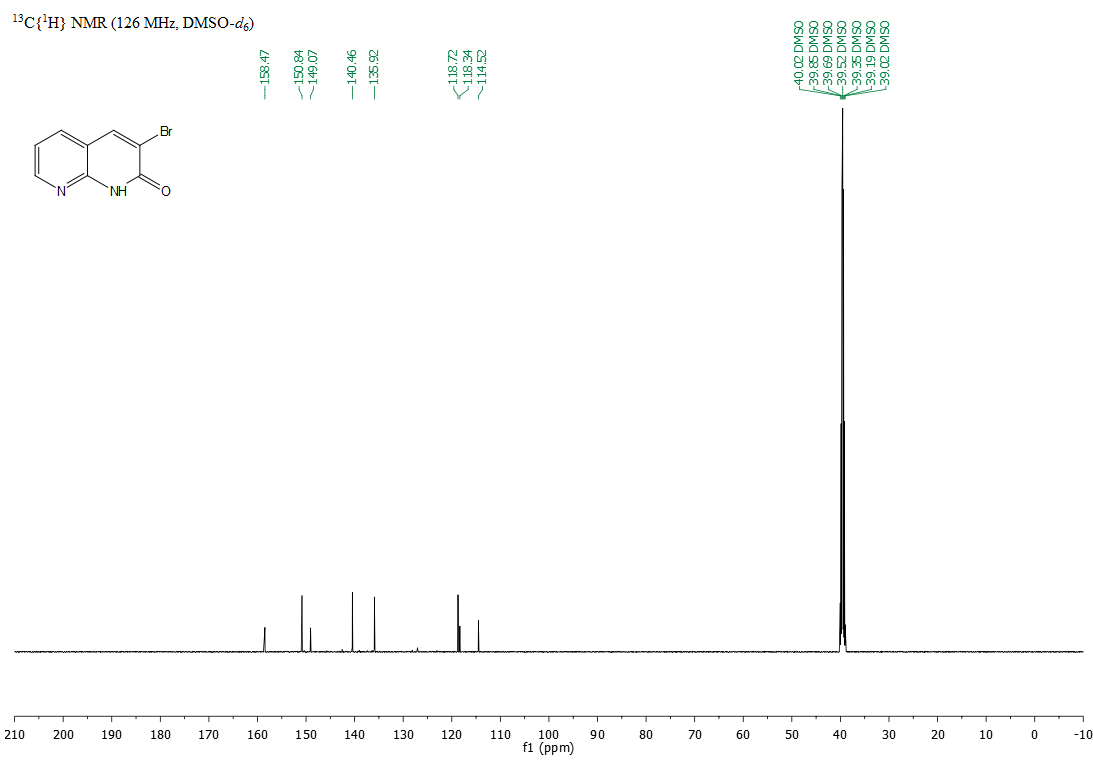


**3-Iodo-1,8-naphthyridin-2(1*H*)-one (5)**


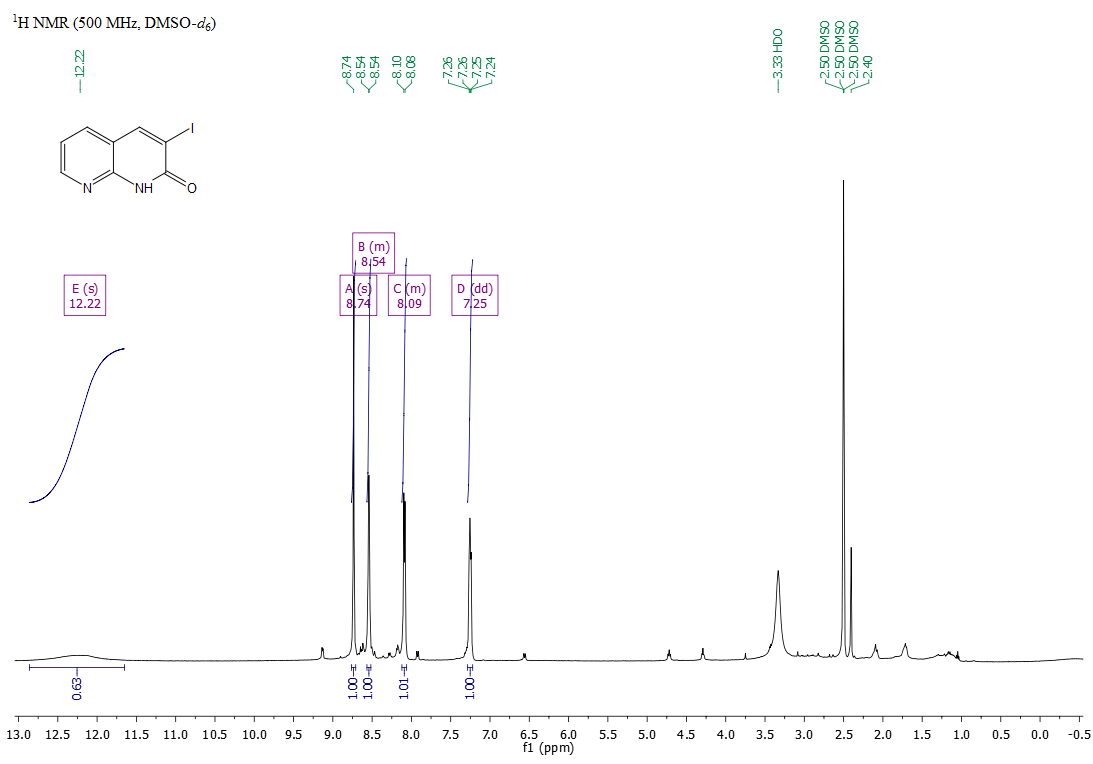


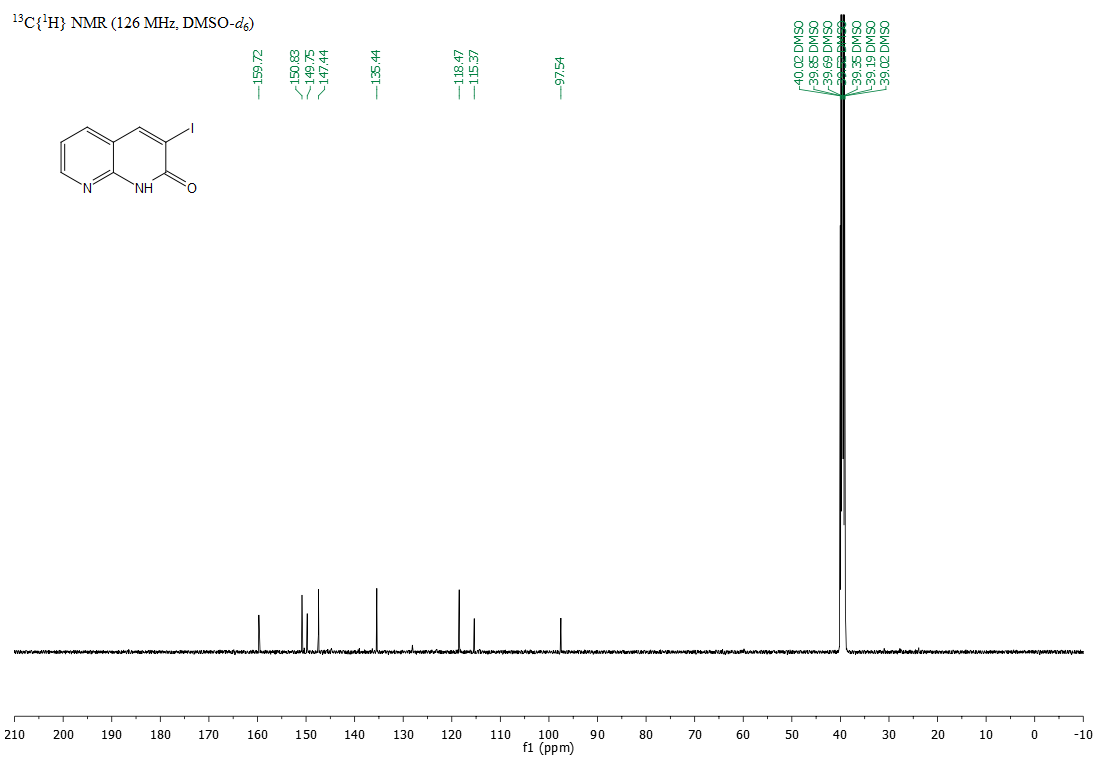


**3-(2'-Deoxy-β-d-ribofuranosyl)-1,8-naphthyridin-2(1*H*)-one (1)**


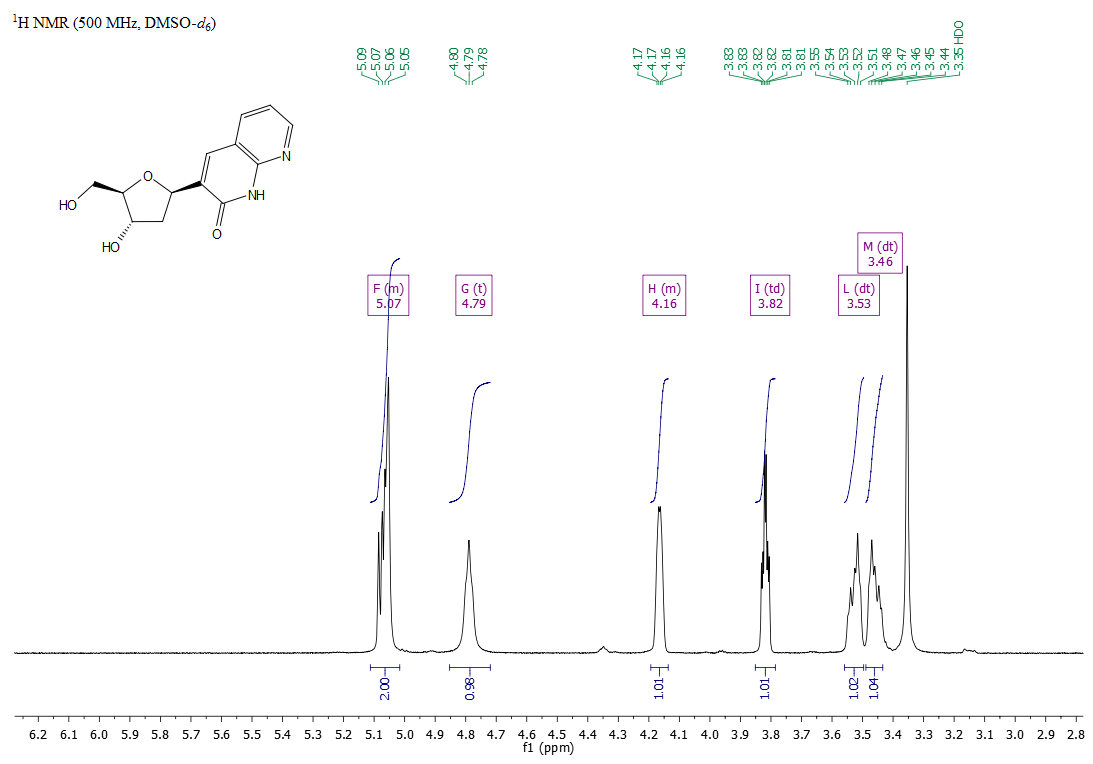


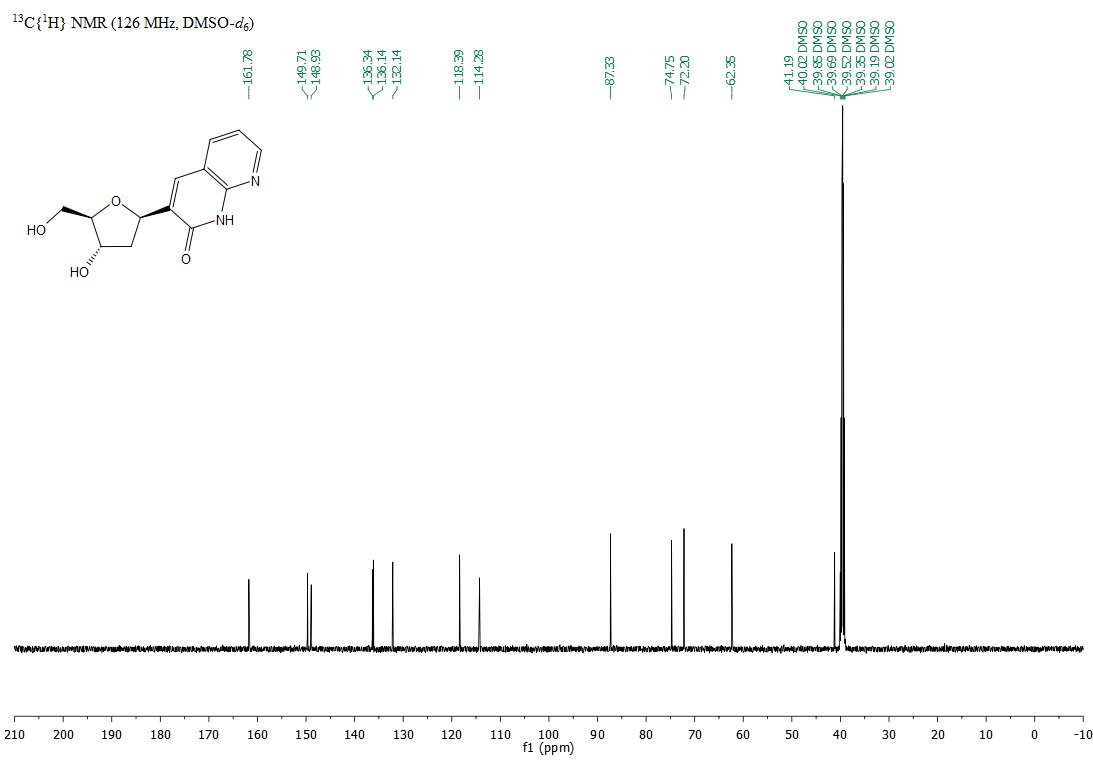


**3-(2'-Deoxy-3'-*O*-[(2-cyanoethyl-*N*,*N*-diisopropyl)phosphoramidyl]-5'-*O*-(4,4'-dimethoxytrityl)-β-d-ribofuranosyl)-1,8-naphthyridin-2(1*H*)-one (2)**


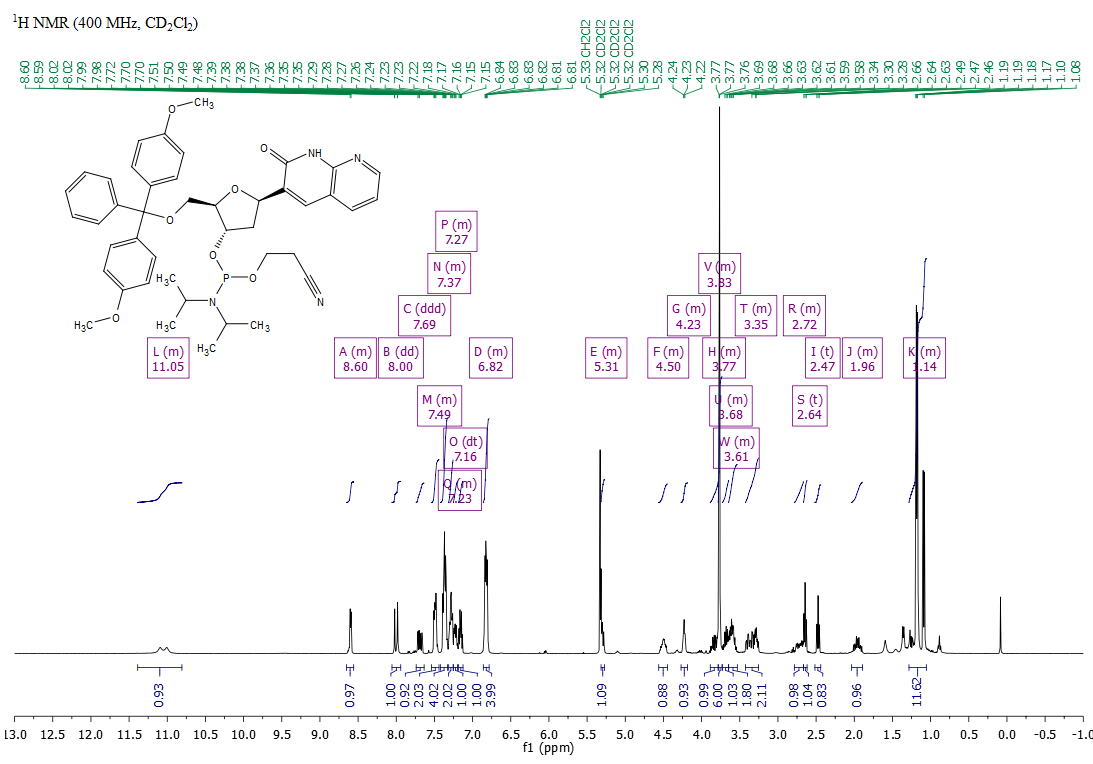


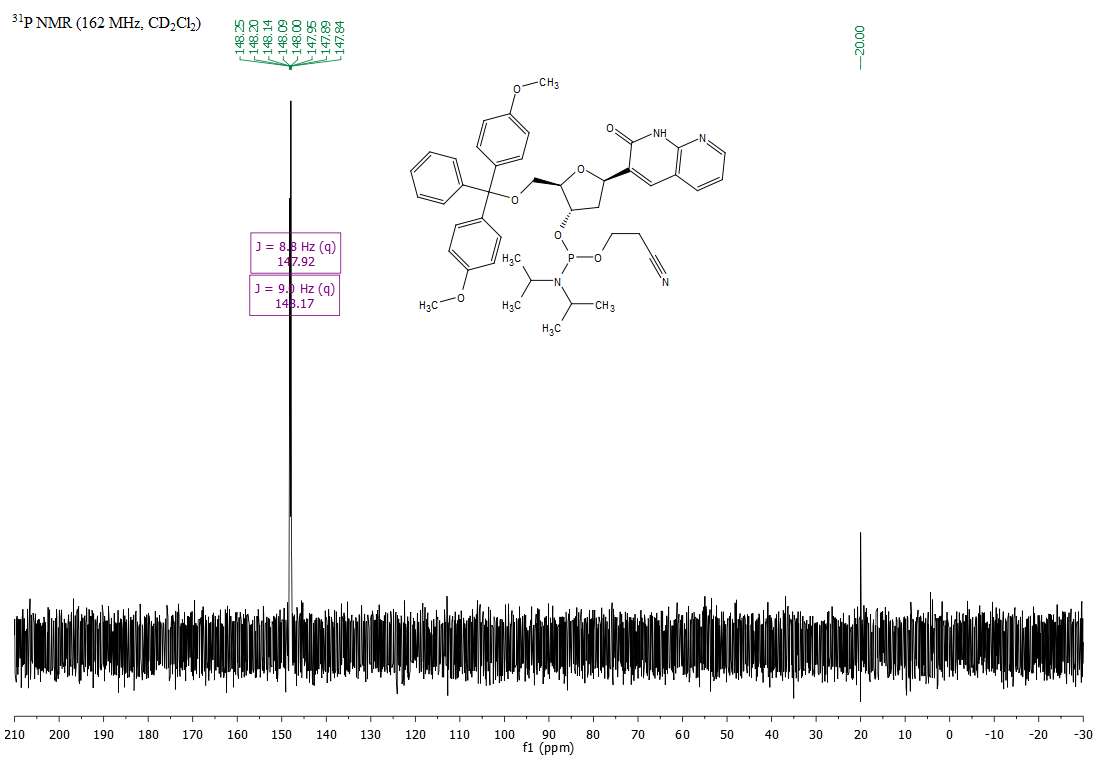


# Additional figures and tables


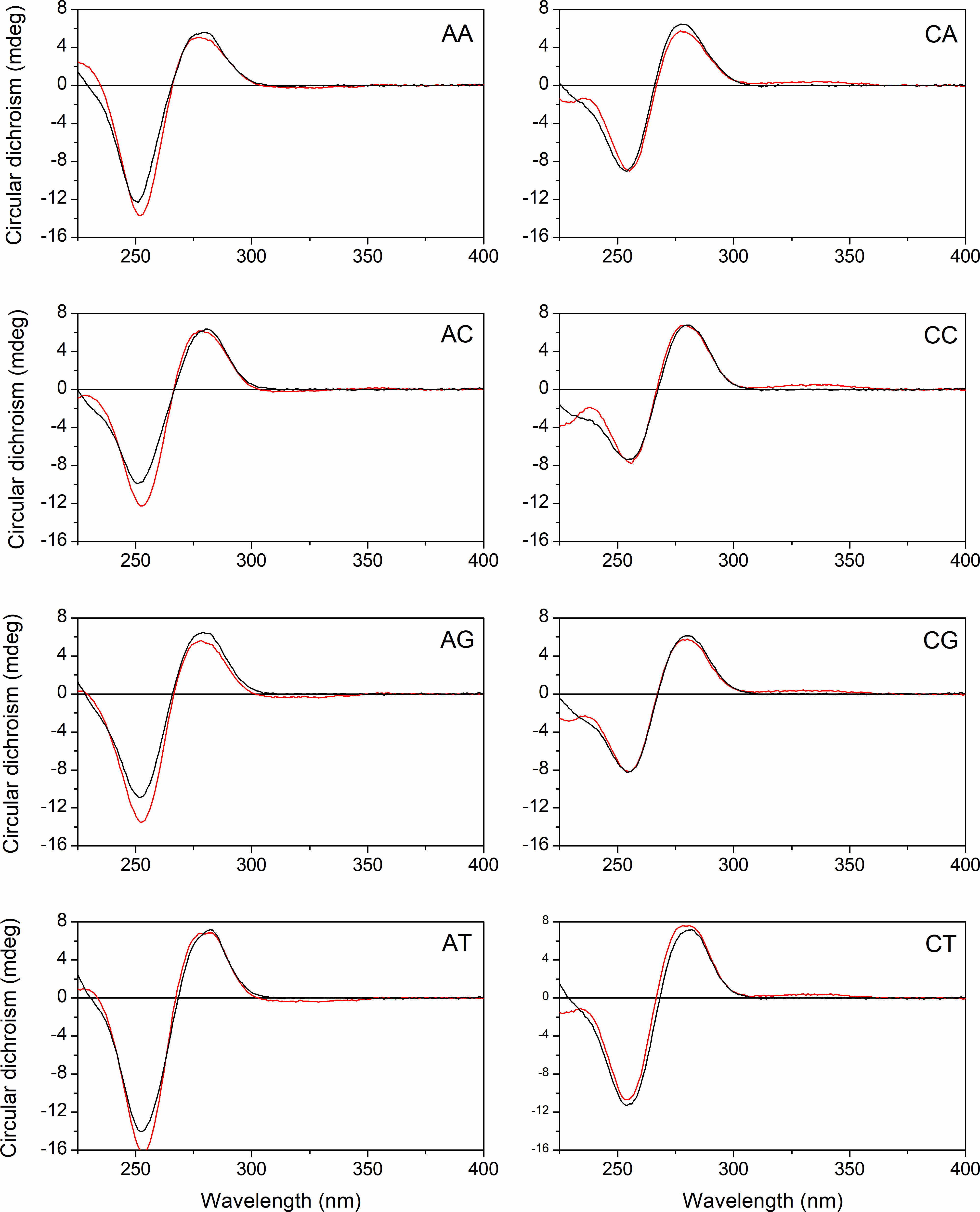


Figure S1. CD spectra of duplexes (AA, AC, AG, AT, CA, CC, CG and CT) with bT (red). Reference spectra of the corresponding unmodified duplexes are shown in black. Duplexes were formed as described in the experimental section and measured at room temperature in phosphate buffer, pH 7.4, 150 mM Na^+^.


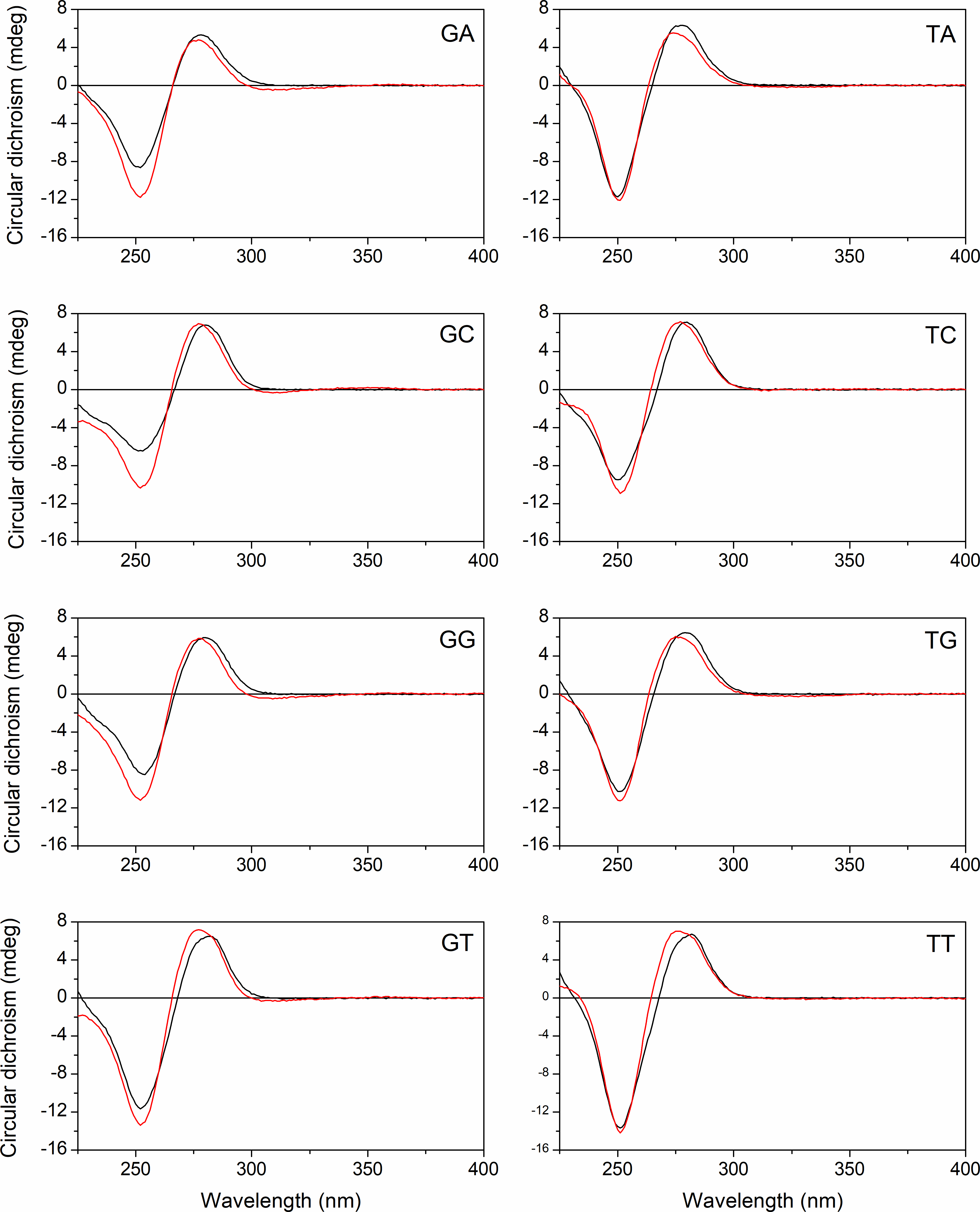


Figure S2. CD spectra of duplexes (GA, GC, GG, GT, TA, TC, TG and TT) with bT (red). Reference spectra of the corresponding unmodified duplexes are shown in black. Duplexes were formed as described in the experimental section and measured at room temperature in phosphate buffer, pH 7.4, 150 mM Na^+^.





Figure S3**.** Absorption (blue) and CD spectra (red) of duplex CC with bT. The reference CD spectra of the corresponding unmodified duplex is shown in black. Duplexes were formed as described in the experimental section and measured at room temperature in phosphate buffer, pH 7.4, 150 mM Na^+^.

Figure S4. Fluorescence melting traces of AA, AC, CT and TT when heated from 20 °C to 85 °C. Samples were measured as described in the experimental section using an excitation wavelength of 325 nm and an emission wavelength of 380 nm.

Table S1. Melting temperatures of bT-modified duplexes (*T*_m_^bT^), unmodified duplexes (*T*_m_^T^), and the difference (Δ*T*_m_) between them with errors.

| Sequence  name^a^ | | DNA sequence^b^ | *T*_m_^bT^  (˚C) | *T*_m_^T^  (˚C) | Δ*T*_m_  (˚C) |
| --- | --- | --- | --- | --- | --- |
| AA | 5′-d(CGCA**A**(bT)**A**TCG)-3′ | | 41.5 ±0.5 | 41.8 ±0.1 | –0.3 ±0.5 |
| AC | 5′-d(CGCA**A**(bT)**C**TCG)-3′ | | 46.2 ±0.2 | 47.0 ±0.3 | –0.8 ±0.4 |
| AG | 5′-d(CGCA**A**(bT)**G**TCG)-3′ | | 47.7 ±0.1 | 48.8 ±0.2 | –1.1 ±0.2 |
| AT | 5′-d(CGCA**A**(bT)**T**TCG)-3′ | | 42.9 ±0.2 | 42.4 ±0.1 | 0.5 ±0.3 |
| CA | 5′-d(CGCA**C**(bT)**A**TCG)-3′ | | 45.8 ±0.1 | 46.4 ±0.2 | –0.6 ±0.2 |
| CC | 5′-d(CGCA**C**(bT)**C**TCG)-3′ | | 49.5 ±0.6 | 50.7 ±0.2 | –1.2 ±0.6 |
| CG | 5′-d(CGCA**C**(bT)**G**TCG)-3′ | | 51.0 ±0.2 | 52.5 ±0.5 | –1.5 ±0.5 |
| CT | 5′-d(CGCA**C**(bT)**T**TCG)-3′ | | 47.3 ±0.2 | 47.9 ±0.1 | –0.6 ±0.3 |
| GA | 5′-d(CGCA**G**(bT)**A**TCG)-3′ | | 44.3 ±0.6 | 44.8 ±0.2 | –0.5 ±0.6 |
| GC | 5′-d(CGCA**G**(bT)**C**TCG)-3′ | | 50.9 ±0.3 | 51.4 ±0.4 | –0.5 ±0.5 |
| GG | 5′-d(CGCA**G**(bT)**G**TCG)-3′ | | 51.0 ±0.3 | 51.0 ±0.3 | 0.0 ±0.4 |
| GT | 5′-d(CGCA**G**(bT)**T**TCG)-3′ | | 47.8 ±0.3 | 48.0 ±0.1 | –0.2 ±0.4 |
| TA | 5′-d(CGCA**T**(bT)**A**TCG)-3′ | | 42.8 ±0.1 | 42.7 ±0.5 | 0.1 ±0.5 |
| TC | 5′-d(CGCA**T**(bT)**C**TCG)-3′ | | 46.6 ±0.2 | 46.4 ±0.3 | 0.2 ±0.3 |
| TG | 5′-d(CGCA**T**(bT)**G**TCG)-3′ | | 48.1 ±0.1 | 48.2 ±0.3 | –0.1 ±0.3 |
| TT | 5′-d(CGCA**T**(bT)**T**TCG)-3′ | | 45.4 ±0.4 | 45.5 ±0.1 | –0.1 ±0.4 |

^a^ Sequences are named by the bases neighbouring bT on the 5′- and 3′-sides, respectively. ^b^ Unmodified samples contain a thymine instead of bT. Duplexes were formed by hybridization with the complementary strand as described in the experimental section. The melting temperatures were calculated as the maximum of the first derivative of the UV-melting curves.

Table S2. Brightness of bT in the 16 modified oligonucleotides in single- (ssDNA) and double-stranded (dsDNA) environment

| Sample^a^ | ssDNA | | | | dsDNA | | | |
| --- | --- | --- | --- | --- | --- | --- | --- | --- |
|  | λ_Abs_  (nm) | ε  (cm^-1^M^-1^) | Φ_F_^b^  (%) | εΦ_F_^b^  (cm^-1^M^-1^) | λ_Abs_  (nm) | ε  (cm^-1^M^-1^) | Φ_F_^b^  (%) | εΦ_F_^b^  (cm^-1^M^-1^) |
| AA | 326 | 11140 | 0.4 | 45 | 328 | 7400 | 0.4 | 29 |
| AC | 325 | 11760 | 0.9 | 103 | 329 | 8620 | 0.3 | 30 |
| AG | 326 | 11500 | 0.2 | 26 | 329 | 9470 | 0.1 | 8 |
| AT | 326 | 11910 | 0.7 | 87 | 328 | 8430 | 0.6 | 48 |
| CA | 325 | 11670 | 1.0 | 116 | 328 | 9620 | 0.3 | 27 |
| CC | 324 | 11900 | 2.2 | 261 | 330 | 8590 | 0.3 | 24 |
| CG | 326 | 11740 | 0.3 | 34 | 330 | 9190 | 0.1 | 8 |
| CT | 324 | 12260 | 2.2 | 267 | 329 | 9360 | 0.3 | 25 |
| GA | 327 | 9290 | 0.2 | 19 | 327 | 7200 | 0.1 | 9 |
| GC | 323 | 12750 | 2.4 | 304 | 330 | 6550 | 0.1 | 10 |
| GG | 326 | 9510 | 0.2 | 18 | 326 | 6560 | 0.1 | 6 |
| GT | 325 | 10230 | 0.5 | 50 | 330 | 6830 | 0.2 | 13 |
| TA | 325 | 12030 | 1.0 | 117 | 329 | 7530 | 0.9 | 66 |
| TC | 325 | 12240 | 1.9 | 239 | 329 | 7320 | 0.5 | 37 |
| TG | 324 | 12070 | 0.3 | 40 | 328 | 7070 | 0.1 | 10 |
| TT | 324 | 12220 | 2.6 | 315 | 330 | 7060 | 1.5 | 104 |

^a^ For sequences, see Table S1. Measurements were performed at room temperature in phosphate buffer, pH 7.4, 150 mM Na^+^, 12.5 mM phosphate. ^b^ Quantum yields were measured with quinine sulphate as reference (Φ_F_ = 54.6% in 0.5 M H_2_SO_4_). The reported values have a standard error ≤ 0.1% and are the averages of two or more measurements.

Table S3. Melting temperatures of bT-modified duplexes (*T*_m_^bT^) measured by absorption and fluorescence, and the difference (Δ*T*_m_) between them.

| Sequence  name^a^ | | DNA sequence | *T*_m_^bT^ -absorption (˚C) | *T*_m_^bT^ -fluorescence (˚C) | Δ*T*_m_  (˚C) |
| --- | --- | --- | --- | --- | --- |
| AA | 5′-d(CGCA**A**(bT)**A**TCG)-3′ | | 41.5 ±0.5 | 45.5 ±0.4 | 4.0 ±0.7 |
| AC | 5′-d(CGCA**A**(bT)**C**TCG)-3′ | | 46.2 ±0.2 | 49.2 ±0.4 | 3.0 ±0.5 |
| CT | 5′-d(CGCA**C**(bT)**T**TCG)-3′ | | 47.3 ±0.2 | 49.0 ±0.1 | 1.7 ±0.3 |
| TT | 5′-d(CGCA**T**(bT)**T**TCG)-3′ | | 45.4 ±0.4 | 46.8 ±0.1 | 1.4 ±0.5 |

^a^ Sequences are named by the bases neighbouring bT on the 5′- and 3′-sides, respectively. ^b^ Duplexes were formed by hybridization with the complementary strand as described in the experimental section. The melting temperatures were calculated as the maximum of the first derivative of the UV/fluorescence-melting curves.

## References

1. Hikishima, S. *et al.* Synthesis of 1,8-Naphthyridine C-Nucleosides and Their Base-Pairing Properties in Oligodeoxynucleotides: Thermally Stable Naphthyridine:Imidazopyridopyrimidine Base-Pairing Motifs. *Angew. Chem.* **117**, 602-604 (2005).
